# Trends in polypharmacy and dispensed drugs among adults in the Netherlands as compared to the United States

**DOI:** 10.1371/journal.pone.0214240

**Published:** 2019-03-22

**Authors:** Monika P. Oktora, Petra Denig, Jens H. J. Bos, Catharina C. M. Schuiling-Veninga, Eelko Hak

**Affiliations:** 1 University of Groningen, University Medical Center Groningen (UMCG), Department of Clinical Pharmacy and Pharmacology, Groningen, The Netherlands; 2 University of Groningen, Groningen Research Institute of Pharmacy, Unit of PharmacoTherapy, -Epidemiology and -Economics, Groningen, The Netherlands; Osakidetza Basque Health Service, SPAIN

## Abstract

**Background and purpose:**

Polypharmacy is becoming increasingly common owing to the ageing population, which can pose problems for patients and society. We investigated the trends in polypharmacy and underlying drug groups among adults in the Netherlands from 1999 to 2014 stratified by age, and compared these with findings from the United States (US).

**Methods:**

We conducted a repeated cross-sectional study using the Dutch IADB.nl prescription database. All patients aged 20 years and older in the period 1999 to 2014 were included. Polypharmacy was defined as the dispensing of five or more chronic drugs at the pharmacological subgroup level. Chi-square tests were applied to calculate the p-value for trends. Changes in prevalences were compared between the Netherlands and the US.

**Results:**

The prevalence of polypharmacy increased from 3.1% to 8.0% (p-value for trend <0.001) over 15 years, and increased in all age groups. The highest rates were observed in patients aged ≥65 years, but the relative increase over time was higher in the younger age groups. Overall, large increases were observed for angiotensin-II inhibitors, statins and proton-pump inhibitors. The relative increase in polypharmacy was larger in the Netherlands than in the US (ratio of polypharmacy prevalence 2.4 versus 1.8). The Netherlands showed larger relative increases for angiotensin-II inhibitors, statins, proton-pump inhibitors, biguanides and smaller relative increases for antidepressants, benzodiazepines and insulins.

**Conclusions:**

Polypharmacy more than doubled from 1999 to 2014, and this increase was not limited to the elderly. The relative increase was larger in the Netherlands compared to the US, which was partly due to larger increases in several guideline-recommended preventive drugs.

## Introduction

Due to ageing populations in many countries, polypharmacy–which is often defined as the chronic use of five or more drugs at the same time–has become more prevalent [1–7]. In the last ten years, the prevalence of polypharmacy has been estimated to range from 10 to 20% in the general population and 40 to 60% in the elderly, partly depending on the definitions and data collection methods used [1,4–6]. Polypharmacy can lead to undesirable consequences for patients and society, including potential drug interactions, drug-related problems, non-adherence, lower quality of life and high costs [8]. These problems are more serious in older patients, notably when they are vulnerable and frail [9,10]. This has led to increasing attention being paid to discontinuing potentially inappropriate medication in older people. On the other hand, polypharmacy is not necessarily inappropriate, since some patients with multimorbidity may need at least five chronic drugs [11].

The prevalence of polypharmacy in the general population increased from 1999 to 2012 on average by 0.2–0.3% per year in Europe and almost 0.6% in the United States [1,4,5]. The baseline frequencies in Europe ranged from 7.7% to 14.1% in 1997–2003 [4–7], whereas this value was 8.2% in the US in 1999–2000 [1]. These increases were higher in the elderly and ranged from 1.3–2.8% per year [1,4–6]. It seems, however, that the increases diminished after 2004–2006 [1,5,6]. Looking at the underlying drugs, the biggest increases were observed for preventive medication, such as cardiovascular drugs and proton-pump-inhibitors (PPIs), whereas decreases were seen for hormone replacement therapy, antibiotics, COX-2 inhibitors [1,4–6]. Nonetheless, patterns differed between countries and time periods. Differences in the healthcare system, drug reimbursement, direct-to-consumer advertising, but also the introduction of new clinical guidelines can play a role in the observed trends in polypharmacy and the underlying drug groups [12–15].

Little is known about the trends in polypharmacy and the underlying drugs in the Netherlands, a country with a relatively strict healthcare system and a conservative prescription policy [16–18]. Population-level dispensing data suggest that polypharmacy increased on average by 0.47% per year in the period 2005–2015 [19]. These data, however, provide no insight into different age groups. Other trend studies have shown that in the Netherlands, between 1996–1999 and 2009–2012, prescription rates of biological agents in children, antidepressants in adults and antibiotics increased, particularly in the elderly [20–22]. Knowledge about trends in polypharmacy, and the age groups and drugs which contribute to these trends, is important to determine whether efforts to reduce polypharmacy may be needed [23].

Our aim is to obtain insights into the trends in polypharmacy stratified by age and the underlying drugs among adults in the Netherlands between 1999 and 2014. The results from this study are compared with the trends observed in the US [1], a country with more liberal healthcare and prescription policies, to determine the extent to which increases in prescription rates in the Netherlands differ from the US.

## Methods

### Study design

We conducted a repeated cross-sectional study to assess trends in polypharmacy and dispensed drugs among adults in the Netherlands from 1 January 1999 until 31 December 2014 using data from a population-based prescription database (IADB.nl). We compared our data with data from the US provided by a study by Kantor *et al*., who evaluated temporal trends in prescription drug use in a repeated cross-sectional survey study among adults in the US from 1999 to 2012 [1].

### Data source

The source population was derived from the University of Groningen IADB.nl, which contains prescription data for more than 20 years from approximately 60 community pharmacies, and covers an estimated population of 600,000 patients. Registration in the database is irrespective of healthcare insurance. Age, gender, and prescription rates among this database population were representative of the Netherlands as a whole, and the database has been widely used for research [24]. Prescription records in the IADB.nl include a patient identifier and contain information on the date of dispensing, the quantity dispensed, the dose regimen, the number of days the prescription was valid, and the Anatomical Therapeutic Chemical code (ATC code) of the dispensed drug [25]. Due to the high patient-to-pharmacy commitment rate in the Netherlands, the medication records for each patient are virtually complete, except for over-the-counter (OTC) drugs that were not on prescription and medication dispensed during hospitalization [24,26]. In this study, we used aggregated data retrieved from the IADB.nl database.

### Study population

The study population comprised of all adults aged 20 years and older included in the IADB.nl database from 1999 to 2014. In line with the previous study by Kantor *et al*., results within the study population were stratified according to age (20–39 years, 40–64 years and ≥65 years old) [1].

### Polypharmacy definition

Polypharmacy was defined in this study as the dispensing of at least five drugs intended for chronic use at the same time [27]. The third level of the ATC Code (ATC3) was used to define pharmacological subgroups, to determine the number of drugs for chronic use. Drugs which had the same ATC3 code were thus considered as one drug, as defined in the national guideline on polypharmacy [27]. Chronic use was when a drug was dispensed for at least 90 days, or at least three times in the period 1 September to 31 December in the year of study [28]. Drugs with no or unknown ATC codes and drugs for topical use, contrast media, radiopharmaceuticals, surgical dressings and general nutrients were excluded (ATC codes starting with D, G01, V, Y, Z) [25].

### Outcomes

The primary outcome was the prevalence of polypharmacy overall and stratified by age (20–39 years, 40–64 years and ≥65 years old). Secondary outcomes included the prevalence of the top 20 dispensed drug groups at the ATC2 (therapeutic subgroup) level, and the prevalence of the drug groups as defined by Kantor *et al*. [1] using the ATC2 and ATC3 (pharmacological subgroup) levels (S1 Table). The prevalence of polypharmacy was calculated by dividing the number of unique patients receiving polypharmacy by the total population in the same year. In contrast to the polypharmacy calculation, all dispensed drugs were included in the secondary outcome calculation. The prevalence of the top 20 dispensed drug groups and the prevalence of the drug groups defined by Kantor were calculated by dividing the number of unique patients receiving this specific drug group by the total study population in the same year. This total number of patients in the denominator was assessed in the September of each year.

### Analysis

The prevalence calculations were conducted on aggregated data retrieved from the IADB.nl database. The prevalences of polypharmacy and the underlying drug groups were averaged for two-year periods and expressed as percentages with 95% Confidence Intervals (CI). Upward trends were defined as increasing prevalence rates for most periods, whereas downward trends were defined as decreasing prevalence rates for most periods. Linear-by-linear association in the Chi-square analysis was used to calculate the p-value for the prevalence rates trend. Statistical significance was assessed at the 2-sided α = 0.05 level. We ranked the prevalences of the drug groups at the ATC2 level to identify the 20 most commonly dispensed drug groups for each two-year period. We calculated the absolute differences and prevalence ratios for these drug groups between the first and the last two-year period. The prevalence ratio indicates the relative increase or decrease in prevalence over the study period. To compare trends in the Netherlands with the US, we calculated the prevalence ratios for polypharmacy and for 18 drug groups–as presented by Kantor *et al*.*–*between 1999–2000 and 2011–2012 [1].

## Results

### Trends in polypharmacy in the Netherlands 1999–2014

**Fig 1:**
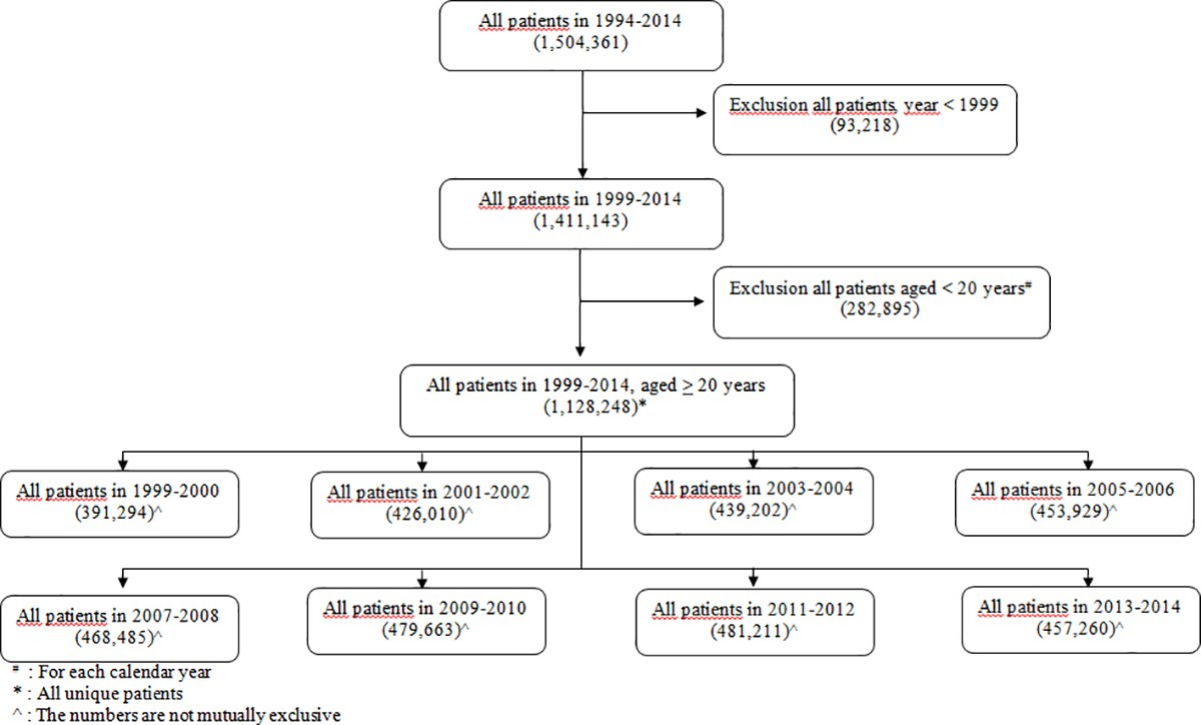
Flow chart of the study population comprising all adults aged 20 years and older present in the IADB.nl database from 1999 to 2014.

The number of patients included in this study from 1999 to 2014 were 391,294, 426,010, 439,202, 453,929, 468,485, 479,663, 481,211 and 457,260, respectively (Fig 1). The populations stratified by age and gender are shown in S2 Table.

**Fig 2:**
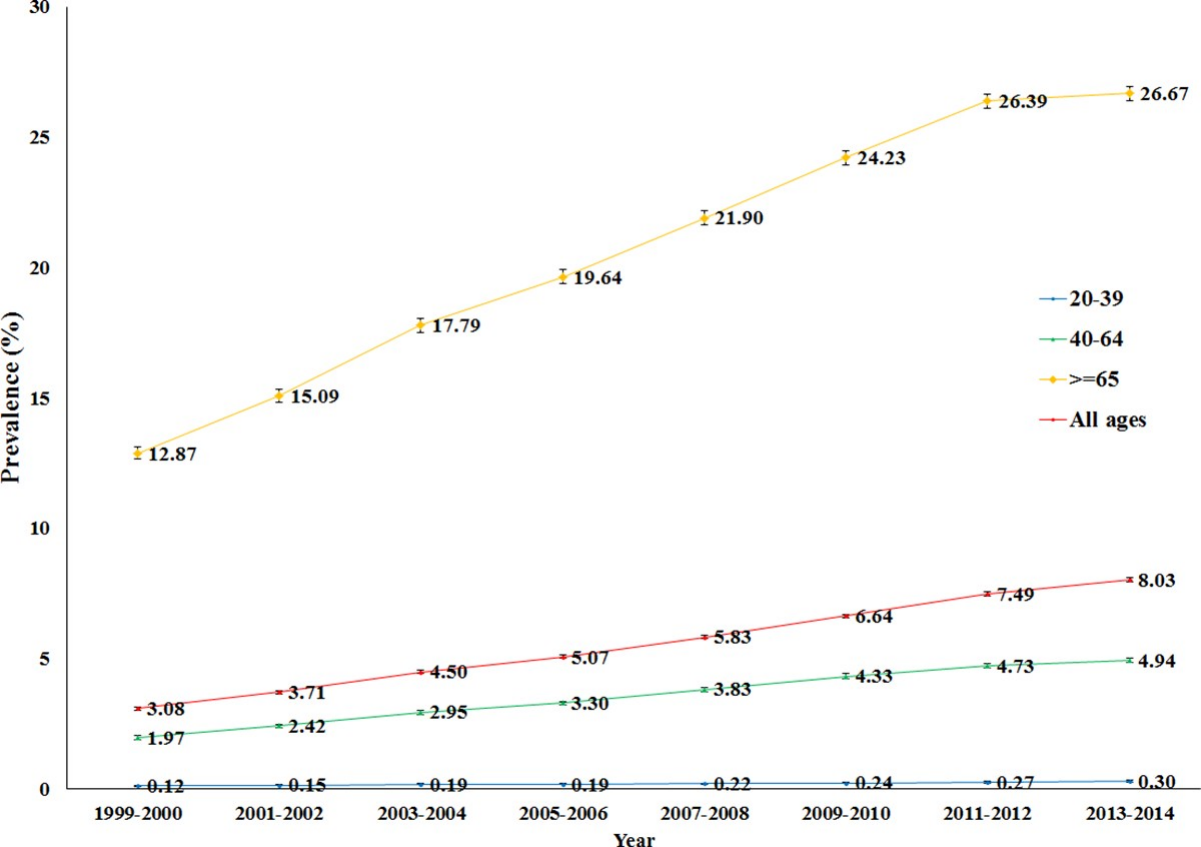
Trends in polypharmacy among adults in the Netherlands 1999–2014, stratified by age.

The two-year polypharmacy prevalence rates increased from 3.08% to 8.03% in the study period (p-value for trend <0.001). When stratified by age group, the prevalence of polypharmacy increased significantly for all age groups (Fig 2 and S3 Table). Adults aged 65 and older showed the highest prevalence compared to the other groups, but the relative increase in the prevalence rate was slightly lower (prevalence ratio 2.07 compared to 2.50 and 2.51 in the other age groups).

### Trends in drug groups in the Netherlands 1999–2014

**Fig 3:**
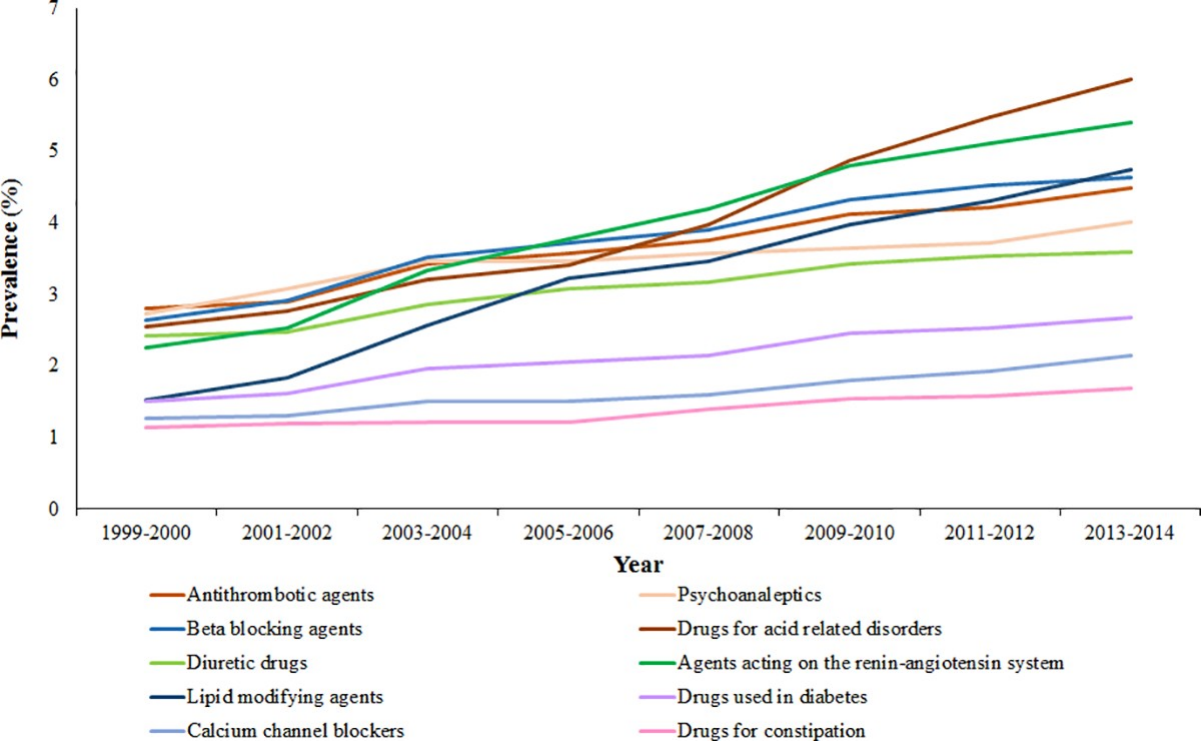
Prevalence of most commonly dispensed drug groups among adults in the Netherlands 1999–2014 (upward trends).

Ten of the therapeutic drug groups (ATC2 level) showed an upward trend from 1999–2000 to 2013–2014, with the most noticeable increases in preventive drug groups (Fig 3 and Table 1). The most prominent increases were observed for drugs for acid-related disorders with an absolute increase of 3.46% (from 2.55% to 6.01%), agents acting on the renin-angiotensin system with an absolute increase of 3.16% (from 2.25% to 5.41%), and lipid modifying agents with an absolute increase of 3.22% (from 1.52% to 4.74%). These drug groups were the top three for absolute and relative increases in prevalence during the study period (Table 1).

**Table 1:** Prevalence of top 20 most commonly dispensed drug groups among adults in the Netherlands 1999–2014 (ranked by descending prevalence ratio).

ATC Code	Therapeutic Subgroup	1999–2000	2001–2002	2003–2004	2005–2006	2007–2008	2009–2010	2011–2012	2013–2014	P for Trend	Difference Prevalence (95% CI)	Prevalence ratio (95% CI)
		n = 391294	n = 426010	n = 439202	n = 453929	n = 468485	n = 479663	n = 481211	n = 457260			
		Prevalence of Use % (95% CI)										
C10	Lipid modifying agents	1.52 (1.48–1.56)	1.83 (1.79–1.87)	2.56 (2.51–2.61)	3.23 (3.18–3.28)	3.46 (3.41–3.51)	3.97 (3.91–4.03)	4.31 (4.25–4.37)	4.74 (4.68–4.80)	< 0.001	3.22 (3.13–3.28)	3.12 (3.01–3.18)
C09	Agents acting on the renin-angiotensin system	2.25 (2.20–2.30)	2.54 (2.49–2.59)	3.34 (3.29–3.39)	3.77 (3.71–3.83)	4.20 (4.14–4.26)	4.80 (4.74–4.86)	5.12 (5.06–5.18)	5.41 (5.34–5.48)	< 0.001	3.16 (3.08–3.24)	2.40 (2.34–2.46)
A02	Drugs for acid related disorders	2.55 (2.50–2.60)	2.77 (2.72–2.82)	3.20 (3.15–3.25)	3.41 (3.36–3.46)	3.98 (3.92–4.04)	4.88 (4.82–4.94)	5.47 (5.41–5.53)	6.01 (5.94–6.08)	< 0.001	3.46 (3.36–3.53)	2.36 (2.30–2.40)
A10	Drugs used in diabetes	1.51 (1.47–1.55)	1.62 (1.58–1.66)	1.96 (1.92–2.00)	2.06 (2.02–2.10)	2.15 (2.11–2.19)	2.45 (2.41–2.49)	2.54 (2.50–2.58)	2.68 (2.63–2.73)	< 0.001	1.17 (1.11–1.23)	1.77 (1.72–1.83)
C07	Beta-blocking agents	2.64 (2.59–2.69)	2.91 (2.86–2.96)	3.52 (3.47–3.57)	3.72 (3.66–3.78)	3.90 (3.84–3.96)	4.33 (4.27–4.39)	4.52 (4.46–4.58)	4.64 (4.58–4.70)	< 0.001	2.00 (1.93–2.08)	1.76 (1.72–1.80)
C08	Calcium channel blockers	1.27 (1.24–1.31)	1.30 (1.26–1.33)	1.50 (1.47–1.54)	1.51 (1.47–1.54)	1.60 (1.56–1.64)	1.79 (1.76–1.83)	1.93 (1.89–1.96)	2.14 (2.10–2.18)	< 0.001	0.87 (0.81–0.92)	1.68 (1.62–1.74)
B01	Antithrombotic agents	2.81 (2.76–2.86)	2.90 (2.85–2.95)	3.42 (3.37–3.47)	3.58 (3.53–3.63)	3.75 (3.70–3.80)	4.13 (4.07–4.19)	4.22 (4.16–4.28)	4.49 (4.43–4.55)	< 0.001	1.68 (1.60–1.76)	1.60 (1.56–1.63)
R01	Nasal preparations	0.80 (0.77–0.83)	0.90 (0.87–0.92)	0.98 (0.95–1.01)	0.92 (0.89–0.95)	1.00 (0.97–1.03)	1.21 (1.18–1.24)	1.21 (1.18–1.24)	1.28 (1.18–1.24)	< 0.001	0.48 (0.44–0.53)	1.60 (1.54–1.68)
C03	Diuretic drugs	2.42 (2.37–2.47)	2.48 (2.43–2.53)	2.86 (2.81–2.91)	3.08 (3.03–3.13)	3.17 (3.12–3.22)	3.43 (3.38–3.48)	3.54 (3.49–3.59)	3.59 (3.54–3.64)	< 0.001	1.17 (1.10–1.25)	1.48 (1.45–1.52)
A06	Drugs for constipation	1.14 (1.11–1.18)	1.20 (1.16–1.23)	1.22 (1.19–1.25)	1.21 (1.18–1.25)	1.40 (1.37–1.44)	1.54 (1.50–1.57)	1.58 (1.55–1.62)	1.69 1,65–1,73	< 0.001	0.55 (0.48–0.61)	1.48 (1.42–1.53)
N06	Psychoanaleptics	2.73 (2.68–2.78)	3.08 (3.03–3.13)	3.47 (3.42–3.52)	3.46 (3.41–3.51)	3.57 (3.52–3.62)	3.65 (3.60–3.70)	3.73 (3.68–3.78)	4.01 (3.95–4.07)	< 0.001	1.28 (1.20–1.35)	1.47 (1.43–1.50)
R03	Drugs for obstructive airway diseases	2.13 (2.08–2.18)	2.29 (2.25–2.33)	2.53 (2.48–2.58)	2.47 (2.42–2.52)	2.50 (2.46–2.54)	2.74 (2.69–2.79)	2.64 (2.59–2.69)	2.68 (2.63–2.73)	< 0.001	0.55 (0.48–0.61)	1.26 (1.22–1.29)
S01	Ophthalmological drugs	1.52 (1.48–1.56)	1.55 (1.51–1.58)	1.72 (1.68–1.76)	1.71 (1.67–1.74)	1.76 (1.72–1.79)	1.82 (1.78–1.86)	1.78 (1.74–1.82)	1.78 (1.74–1.82)	< 0.001	0.26 (0.20–0.31)	1.17 (1.13–1.21)
J01	Antibacterial drugs	2.69 (2.64–2.74)	2.68 (2.63–2.73)	2.96 (2.91–3.01)	2.97 (2.92–3.02)	2.98 (2.93–3.03)	3.23 (3.18–3.28)	2.80 (2.75–2.85)	2.73 (2.68–2.78)	< 0.001	0.04 (0.03–0.11)	1.01 (0.99–1.04)
N02	Analgesic drugs	2.31 (2.26–2.36)	2.35 (2.30–2.40)	2.33 (2.29–2.37)	1.96 (1.92–2.00)	2.04 (2.00–2.08)	2.17 (2.13–2.21)	2.26 (2.22–2.30)	2.21 (2.17–2.25)	< 0.001	-0.10 (-0.16)- (-0.04)	0.96 (0.93–0.98)
D07	Topical dermatological corticosteroids	1.71 (1.67–1.75)	1.69 (1.65–1.72)	1.77 (1.73–1.81)	1.74 (1.70–1.78)	1.61 (1.57–1.65)	1.65 (1.61–1.68)	1.52 (1.48–1.55)	1.50 (1.46–1.54)	< 0.001	-0.21 (-0.27)—(-0.16)	0.88 (0.85–0.90)
C01	Cardiac therapy	1.30 (1.26–1.33)	1.19 (1.16–1.23)	1.22 (1.18–1.25)	1.05 (1.02–1.08)	0.99 (0.96–1.01)	0.98 (0.96–1.01)	0.96 (0.93–0.99)	0.97 (0.94–1.00)	< 0.001	-0.33 (-0.37)-(-0.28)	0.75 (0.72–0.77)
N05	Psycholeptics drugs	6.32 (6.24–6.40)	6.12 (6.05–6.19)	6.29 (6.22–6.36)	5.83 5.76–5.90)	5.52 (5.45–5.59)	4.73 (4.67–4.79)	4.56 (4.50–4.62)	4.56 (4.50–4.62)	< 0.001	-1.76 (-1.86)-(-1.66)	0.72 (0.71–0.73)
M01	Anti-inflammatory and antirheumatic drugs	3.51 (3.45–3.57)	3.53 (3.47–3.59)	3.64 (3.58–3.70)	2.98 (2.93–2.03)	2.73 (2.68–2.78)	2.71 (2.66–2.76)	2.47 (2.43–2.51)	2.31 (2.27–2.35)	< 0.001	-1.20 (-1.27)-(-1.13)	0.66 (0.64–0.67)
G03	Sex hormones and modulators of the genital system	3.35 (3.29–3.41)	3.10 (3.05–3.15)	2.94 (2.89–2.99)	2.48 (2.43–2.53)	2.25 (2.21–2.29)	2.30 (2.26–2.34)	2.13 (2.09–2.17)	2.00 (1.96–2.04)	< 0.001	-1.35 (-1.42)-(-1.28)	0.60 (0.58–0.61)

**Fig 4:**
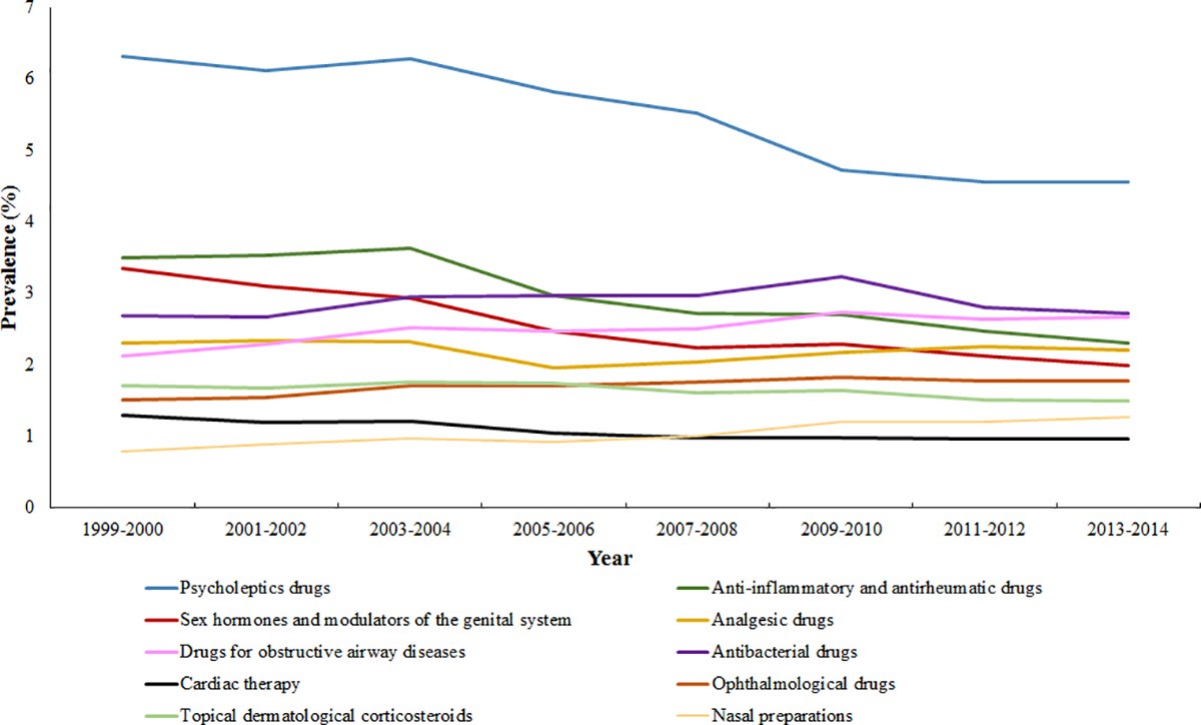
Prevalence of most commonly dispensed drug groups among adults in the Netherlands 1999–2014 (constant or downward trends).

Four drug groups showed downward trends (Fig 4). These included psycholeptic drugs with an absolute decrease of 1.76% (from 6.32% to 4.56%), anti-inflammatory and anti-rheumatic drugs with an absolute decrease of 1.20% (from 3.51% to 2.31%), sex hormones and genital system modulators with a absolute decrease of 1.35% (from 3.35% to 2.00%), and cardiac therapy with an absolute decrease of 0.33% (from 1.30% to 0.97%). The rates for the six remaining drug groups remained fairly stable or fluctuated over time. For example, antibacterial dispensing increased up to 2009–2011, after which it decreased to the levels observed in 1999–2000 (Fig 4 and Table 1).

Both the increasing and decreasing trends showed significance, also in the lower pharmacological subgroups (Table 1). For example, the increase in drugs for acid-related disorders was paralleled by a signicant increase in proton-pump inhibitors. In some cases, not all of the underlying drugs showed increases. For example, of the lipid modifying agents, the most significant increases were observed for statins. Diverse patterns were observed for some groups. For example, for analgesics, downward trends were observed for NSAIDs and salicylates, an upward trend for narcotic analgesics, and a temporary increase for COX-2 inhibitors (S4 Table).

### Comparison of trends with the United States

The two-year polypharmacy prevalence rates from 1999 to 2012 in the US, as presented by Kantor *et al*., were 8.2%, 11%, 14%, 14%, 15%, 14% and 15% respectively, which represent an increase of 0.57% per year.^1^ The prevalence rates observed in the Netherlands ranged from 3.1% to 7.5% in the same period, which represent an increase of 0.37% per year. In the US, the prevalence appeared to level off after 2003–2004, whereas it consistently increased for the overall population in the Netherlands (Fig 2).

In the US, this pattern was partly driven by the 40–64 age group, for whom the polypharmacy prevalence peaked at 15.1% in 2003–2004, whereas the prevalence rate peaked at 40.4% in 2007–2008 for the 65 and older age group. In the Netherlands, increases were observed for all age groups until 2011–2012, after which the increase appeared to level off in the 65 and older group (Fig 2). The age and gender distribution were similar for the US and the Netherlands in 2011–2012. The percentages for the 20–39, 40–64 and ≥65 age strata in the US were 35.3%, 42.3%, and 22.5%, respectively, while in the Netherlands they were 35.3%, 44.7% and 20.0%, respectively. The population in the US was 49.3% males and 50.7% females, whereas this was 48.2% males and 51.8% females in the Netherlands.

The prevalence rates for all drug classes in the Netherlands were consistently lower than in the US during the whole study period (S5 Table and Table 2 in Kantor *et al*.). Antihypertensive agents were the most prevalent drugs in both countries, and increases in prevalence were observed for almost all antihypertensive subgroups. However, the relative increase for angiotensin-II inhibitors was larger in the Netherlands compared to the US (prevalence ratio 4.4 versus 2.7). The prevalence of antihyperlipidemic agents, in particular statins, also increased in both countries. Again, the relative increase for statins was larger in the Netherlands than in the US (prevalence ratio 2.8 versus 2.5). Similar patterns were seen for proton-pump inhibitors (prevalence ratio 3.6 in the Netherlands and 2.0 in the US) and thyroid hormones (prevalence ratio 2.0 in the Netherlands and 1.2 in the US). On the other hand, the relative increases for antidepressants and benzodiazepines were larger in the US than in the Netherlands (prevalence ratio 1.9 versus 1.4, and 1.4 versus 0.6, respectively). The pattern was mixed for antidiabetic drugs, with a larger relative increase for biguanides in the Netherlands (prevalence ratio 3.7 versus 2.7) and a larger relative increase for insulins in the US (prevalence ratio 2.3 versus 1.5). The relative decreases in sex hormones and contraceptives were similar in both countries. Of note, where the prevalence of COX-2 inhibitors slightly increased in the Netherlands, it decreased in the US over the study period (prevalence ratio 2.1 versus 0.3).

## Discussion

### Principal findings and their relationship with the literature

The prevalence of polypharmacy among adults in the Netherlands between 1999 and 2014 increased more than twofold, from around 3% to 8% in the overall population. Increases were observed in all age groups, but the relative increases were larger in the under-65 age groups. Preventive therapies showed the highest prevalence rates, and the largest increases were seen for proton-pump inhibitors, angiotensin-II inhibitors and statins. In general, the increase in prevalence rate was lower in the Netherlands compared to the US, but similar trends were observed for many drugs. Polypharmacy prevalence showed a steady increase up to 2012 in the Netherlands, whereas it seemed to peak in the US in 2007. The Netherlands showed larger relative increases than the US for angiotensin-II inhibitors, statins, proton-pump inhibitors, biguanides, and smaller relative increases for antidepressants, benzodiazepines and insulins.

Our findings confirm that polypharmacy is common in the elderly population and has increased over time, as reported in other studies, despite some differences in time and setting [1,4–7]. The increasing trends of polypharmacy prevalence have also occurred in some other Europe countries, such as Ireland, Sweden, Switzerland, and the United Kingdom [4–7]. Ageing is an important factor which drives the development of polypharmacy [1,4]. Living longer increases the manifestation of morbidity and chronic illness [29], leading to the use of multiple drugs. In 2012, the average life expectancy in the Netherlands reached 81.2 years [30]. Particular attention should be paid to polypharmacy because it is associated with many drug-related problems, such as adverse drug events (ADEs), drug-drug interactions, medication non-adherence, functional decline and cognitive impairment [8,31]. Moreover, the development of polypharmacy–also in the younger age groups- contributes to the increase in healthcare costs for both the patient and society [32].

Several explanations can be offered for the increases observed for specific drugs in our study. Proton-pump inhibitors (PPIs) were the main group responsible for the increase in the prescription rate for drugs for acid-related disorders. At the beginning of the study period in the Netherlands, PPIs were considered as second-stage treatments for minor gastric disorders. The increase in their use has become a matter for concern [33]. Nevertheless, PPIs were the key prescription drug group for acid-related disorders in this period because other drugs for acid-related disorders were all OTC medication. PPIs have been recommended in helicobacter eradication protocols since 1999. Furthermore, gastric protection using PPIs to counter or alleviate gastric problems caused by nonsteroidal anti-inflammatory drugs (NSAID) and low-dosed salicylates received more attention during the study period, also reflected in the update of the clinical guideline for gastric complaints in 2013 [34].

A general increase was observed for preventive cardiovascular therapies, including ACE-inhibitors, angiotensin-II inhibitors, β-blockers and statins. Clinical guidelines for cardiovascular risk management advocated proactive screening for cardiovascular risks [35,36], which has undoubtedy resulted in increasing numbers of patients being treated with such first-line treatments [37,38]. The Dutch guidelines were updated in 2006, and recommendations became more stringent regarding the risk factor thresholds for requiring treatment with antihypertensives and/or statins, particularly for patients with diabetes [36,39]. ACE-inhibitors and angiotensin-II inhibitors were recommended for these patients in particular, because of their additional renal protective effects.

Some drugs showed a downward trend during the study period. The dispensing of psycholeptics, in particular benzodiazipines, decreased substantially between 1999 and 2014. This may partly be due to a restriction in the reimbursement offered for benzodiazepines in the Netherlands, which started in 1 January, 2009. This was aimed at restricting chronic benzodiazepine use to patients with certain indications (e.g. epilepsy, specific psychiatric disorders and palliative care). Our study, however, shows that the trend towards fewer benzodiazepine prescriptions had already begun in 2005. The prescription of anti-inflammatory and anti-rheumatic drugs, in particular NSAIDs, also declined. NSAIDs can be prescribed in the Netherlands but it is estimated that one-third of the people use OTC NSAIDs, which are not included in our study [40]. This implies that the reduction in dispensed NSAIDs may be mirrored by an increase in OTC NSAID use.

Another notable result concerns the use of antibacterial drugs. These drugs showed a temporary increase in 2009–2010. This increase may relate to an influenza pandemic in Europe in that period, which involved a new strain of influenza, subtype H1N1. The first such case was diagnosed on 30 April 2009 in the Netherlands [41]. Antibiotics were prescribed as the treatment for secondary bacterial infections caused by the complications of influenza [42]. The use of antibiotics decreased from 2011. This has also been observed in other European countries, such as Finland and Sweden [43]. This may be in part be due to a European strategic action plan on antibiotic resistance issued in 2011 by WHO Europe, and supported by the European Centre for Disease Prevention and Control (ECDC) [44].

Increases in prevalence rates of polypharmacy across all age groups and prevelance rates for the underlying drugs were clearly lower in the Netherlands than in the US. This may in part reflect the substantial differences in healthcare systems and policies between the two countries. It is well-known that the Netherlands spends significantly less on healthcare per capita than the US, although the healthcare outcomes in the Netherlands are similar to or even better than in the US [45,46]. The majority of healthcare costs in the Netherlands are covered by a compulsory basic health insurance. This policy provides for a degree of equity among the population, which is likely to result in efficient healthcare use [12]. Conversely, the US has a mixed system of private coverage, cost sharing and publicly-funded coverage through various programmes. The healthcare and insurance systems in the US are more market oriented [15]. Furthermore, the Netherlands have a gatekeeping system and do not allow direct-to-consumer advertising for prescription drugs. In contrast, patients in the US are widely exposed to prescription drug advertising and are allowed to go directly to a specialist, which may leed to higher prescription rates [14,15].

Beyond healthcare system differences, differences in the population may also explain the results. First, the population in the US varies more in race and ethnicity than in the Netherlands [1]. This factor may influence the use of prescription drugs. Second, there is an epidemic of obesity in the US, and polypharmacy is more common in obese patients than in people with a normal BMI [1]. Obese patients with type 2 diabetes are more likely to need insulin, which may explain the differences in the relative increases in the use of antidiabetic agents between the Netherlands and the US. Finally, there may be differences in the attention to or timing of specific issues. For example, COX-2 inhibitors were never very popular in the Netherlands, and therefore the fact that rofexocib was taken off the market in 2004 only had a small impact on the overall trend, whereas this was regarded as an important factor for explaining the downward trend of COX-2 inhibitors in the US [1].

Of note is the leveling observed in the polypharmacy prevalence in the US after 2004. Kantor *et al*. suggest that this may reflect a saturation of the market and could be related to policy changes or a downward trend in direct-to-consumer advertising after the mid-2000s. In the Netherlands, the prevalence of polypharmacy appeared to peak in the 65-years-and-older age group by the end of our study period. This may be the result of increased attention being paid to polypharmacy in the vulnerable elderly, which is also reflected in the Dutch polypharmacy in the elderly guideline published in 2012 [27]. Recent population level data confirm that the increase in polypharmacy seems to have been leveling off in the Netherlands since 2015 [47].

### Study strengths and limitations

This study relies on data collected by the IADB.nl prescription database which provides a complete record of dispensed drugs over time in the covered population. This database is representative of the Dutch population. Moreover, information bias did not occur because this study indirectly examines the medication used in the study population.

Some limitions need to be mentioned. First, information about the actual use of drugs by the patients was unavailable because we only looked at the drug prescriptions being dispensed. We do not know whether patients actually took the drugs they were prescribed. We also do not know which OTC drugs the patients used. In the Netherlands, OTC drugs include drugs for common complaints, such as pain, cough, hay fever, itching, and stomach complaints. Second, for the polypharmacy calculation we counted drugs at the pharmacological group level (ATC3) in the last four months of each year, as proposed in the Dutch guidelines for polypharmacy. This assumes that drugs within this level are similar and usually not combined for chronic use. This prevents switches between drugs at this level in the assessment period from being double counted. Combination drugs at this level, however, were counted as a single drug, which could cause underestimation of the polypharmacy prevalence. Third, we used aggregated data when testing for trends and were therefore unable to adjust for correlations at patient level over time. Different methods were used for estimating prescription rates in our study as compared to the US study. In the US study, information about prescription drug use was collected from household interviews, where patients were asked to show the prescription drugs that they had taken in the preceding 30 days. This represents the actual drug use, but might underestimate drug use if patients do not remember or reveal all the medication they took. In our study, we collected data from a prescription database which might overestimate drug use, since patients might not actually take all the medication dispensed to them. An earlier study by Sediq *et al*. showed that the concordance of information collected from interviews and from the prescription database is generally good for chronically used medication [26].

### Implication for policy and practice

Our study showed that polypharmacy prevalence increased by 1% every three years in the overall population. This is a substantial rise given the initial prevalence of 3% in the period 1999–2001. As mentioned, polypharmacy in itself does not reflect medication inappropriateness, although it is an indicator of medication burden and possible drug-related problems [8,9,48–50]. Considering trends in the underlying drug groups provides some insight into the potential appropriateness. The increased prevalences observed for several preventive drug groups appears to be in line with changes in clinical practice and the guidelines applied in the Netherlands, in particular regarding cardiovascular risk management. Increases and decreases seem to relate to changes in the policies for certain drugs, such as in the reimbursement system or prescription policy. Some trends, however, might be worrying. Where strict management of cardiovascular risk factors is advocated in general, concerns are growing about overtreatment in the elderly population [51]. The increase in these drugs may be appropriate in the groups aged under 65, on the other hand. More detailed analyses are thus warranted in this younger population. Commonly used tools, such as the START/STOPP criteria, have been developed for people aged 65 and older. Therefore, there is a need for similar criteria which can be applied in younger age groups. For example, following from our results, this could include potentially inappropriate use of PPIs. New reports about serious adverse events have informed more critical views regarding the increasing use of these drugs and indications for chronic use in the younger age groups are limited [34,52].

Complex interventions are needed to manage polypharmacy and halt rising trends in the use of potentially inappropriate drugs, such as advanced medication reviews and medication deprescribing [23]. Constant monitoring of prescription drug use is warranted to assess the effects of policy measures and signal potential inappropriate drug use.

## Conclusion

The prevalence of polypharmacy in the Netherlands has increased significantly from 1999 to 2014. This increase was not limited to the elderly, and may even have halted in the elderly in 2013–2014. Increases in the underlying drugs partly relate to changed guideline recommendations and reimbursement policies, but this does not exclude the possibility of overtreatment, particularly in the elderly. Despite differences in the methods used to assess prescription drug use, increases in prevalence rates are lower in the Netherlands than in the US. Over time the differences in prevalence rates have become smaller.

## Supporting information

S1 Table Adjustment of ATC codes according to the top 18 drug groups in the United States 1999–2012. Kantor et al. used nationally representative data from the National Health and Nutrition Examination Survey (NHANES) to estimate the prevalence of prescription drug use in the United States from 1999 to 2012. They observed significant increases in both the overall prescription drug use and polypharmacy. All the drugs were coded according to the Multum Lexicon Therapeutic Classification Scheme. In this study, these drug groups were approximated using the Anatomical Therapeutic Chemical (ATC) classification system.
(DOCX)

S2 Table Number of population stratifed by age and gender in IADB 1999–2014. (DOCX)

S3 Table Numbers for population, polypharmacy and polypharmacy prevalence, 1999–2014, stratifed by age. (DOCX)

S4 Table Trends in prescription drug use among adults in the Netherlands 1999–2014. Abbreviations: ACE, angiotensin-converting enzyme; COX-2, cyclooxygenase 2; NA, not applicable; NSAID, nonsteroidal anti-inflammatory drug; SSNRI, selective serotonin–norepinephrine reuptake inhibitor; SSRI, selective serotonin reuptake inhibitor. ^a^Subgroups are presented in the order of Kantor *et al*. as 18 drugs classes from 1999–2012 and adjusted with the ATC codes system (see S1 Table). ^b^Excludes COX-2 inhibitors.
(DOCX)

S5 Table Prevalence of prescription drug use among adults in the Netherlands. Abbreviations: ACE, angiotensin-converting enzyme; COX-2, cyclooxygenase 2; NA, not assessed; NSAID, nonsteroidal anti-inflammatory drug; SSNRI, selective serotonin–norepinephrine reuptake inhibitor; SSRI, selective serotonin reuptake inhibitor.^a^Subgroups are presented in the order of Kantor *et al*. as 18 drugs classes from 1999–2012 and adjusted with the ATC codes system (see S1 Table). ^b^Excludes COX-2 inhibitors.
(DOCX)

S6 Table STROBE statement. (DOCX)
