# Supplementary material for: Trends in polypharmacy and dispensed drugs among adults in the Netherlands as compared to the United States
Source: PLoS One. 2019 Mar 22;14(3):e0214240. doi: 10.1371/journal.pone.0214240 (PMC6430511; doi:10.1371/journal.pone.0214240)
Supplement: S1 Table — Kantor et al. used nationally representative data from the National Health and Nutrition Examination Survey (NHANES) to estimate the prevalence of prescription drug use in the United States from 1999 to 2012. They observed significant increases in both the overall prescription drug use and polypharmacy. All the drugs were coded according to the Multum Lexicon Therapeutic Classification Scheme. In this study, these drug groups were approximated using the Anatomical Therapeutic Chemical (ATC) classification system. (DOCX) [file pone.0214240.s001.docx]

**S1 Table. Adjustment of ATC Codes according to the top 18 drug groups in the United States 1999-2012^a^**

| **No** | **Subgroups** | **ATC Codes** |
| --- | --- | --- |
| 1 | Antihypertensive agent   - Antihypertensives (other) - Angiotensin-converting enzyme (ACE) inhibitors - Angiotensin II inhibitors - β-blockers (cardioselective and noncardioselective) - Calcium-channel blockers - Any diuretic (loop, potassium-sparing, thiazide) - Anti-hypertensive combination | - C02 - C09A, C09B - C09C, C09D - C07 - C08 - C03 (C03C, C03D, C03A) - C02L, C02N |
| 2 | Antihyperlipidemic agents   - Fibric acid derivatives - Statins - Antihyperlipidemic combinations | C10   - C10AB - C10AA - C10B |
| 3 | Antidepressants   - Phenylpiperazine - SSNRIs - SSRIs - Tricyclics | N06A   - Not Applicable - N06AX - N06AB - N06AA |
| 4 | Prescription analgesics   - COX-2 inhibitors - Narcotic analgesics - Prescription NSAIDs (exclude COX-2 inhibitors) - Salicylates - Miscellaneous analgesics | N02   - M01AH - N02A - M01A (excl. M01AH) - N02BA - N02B |
| 5 | Hormones   - Sex - Contraceptives - Noncontraceptives | G03   - G03B, G03C, G03D, G03E, G03G, G03H, G03X - G03A - G03F |
| 6 | Antidiabetic agents   - Biguanides - Insulin - Sulfonylureas - Thiazolidinedions | A10   - A10BA - A10A - A10BB - A10BG |
| 7 | Prescription proton-pump inhibitors (PPIs) | A02BC |
| 8 | Thyroid hormones | H03AA |
| 9 | Anxiolytics, sedatives, hypnotics   - Benzodiazepines | N05   - N05BA, N05CD, N05CF |
| 10 | Anticonvulsants   - Benzodiazepines - γ-aminobutyric acid analogs | N03   - N03AE - N03AX |
| 11 | Bronchodilators   - Sdrenergic bronchodilators - Anticholinergic bronchodilators - Bronchodilators combinations | R03   - R03A, R03C - R03BB - R03AH, R03AK, R03AL |
| 12 | Antibiotics   - Oral antibiotics | J01 |
| 13 | Antiarrhythmic agents   - Class I and III - Class IV - Class V | - C01B - C08DA01, C08DB01 - C01EB10, C01AA |
| 14 | Coagulation modifiers   - Anticoagulants (warfarin) - Antiplatelet agents - Clopidogrel | B01, B02   - B01 (B01AA03) - B01AC - B01AC04 |
| 15 | Muscle relaxant | M03 |
| 16 | Nasal preparations: nasal steroids | R01   - R01AD |
| 17 | H_2_ antagonists | A02BA |
| 18 | Prescription antihistamines | R060. |

**^a^** Kantor *et*^a^Kantor *et al.* used nationally representative data from the National Health and Nutrition Examination Survey (NHANES) to estimate the prevalence of prescription drug use in the United States from 1999 to 2012. They observed significant increases in both the overall prescription drug use and polypharmacy. All the drugs were coded according to the Multum Lexicon Therapeutic Classification Scheme. In this study, these drug groups were approximated using the Anatomical Therapeutic Chemical (ATC) classification system.
