# Supplementary material for: Trends in polypharmacy and dispensed drugs among adults in the Netherlands as compared to the United States
Source: PLoS One. 2019 Mar 22;14(3):e0214240. doi: 10.1371/journal.pone.0214240 (PMC6430511; doi:10.1371/journal.pone.0214240)
Supplement: S2 Table — (DOCX) [file pone.0214240.s002.docx]

|  | **1999-2000** | | **2001-2002** | | **2003-2004** | | **2005-2006** | | **2007-2008** | | **2009-2010** | | **2011-2012** | | **2013-2014** | |
| --- | --- | --- | --- | --- | --- | --- | --- | --- | --- | --- | --- | --- | --- | --- | --- | --- |
|  | **No. of patients** | **%** | **No. of patients** | **%** | **No. of patients** | **%** | **No. of patients** | **%** | **No. of patients** | **%** | **No. of patients** | **%** | **No. of patients** | **%** | **No. of patients** | **%** |
| **Overall** | 391294 |  | 426010 |  | 439202 |  | 453929 |  | 468485 |  | 479663 |  | 481211 |  | 457260 |  |
| **Age group (year)** | |  |  |  |  |  |  |  |  |  |  |  |  |  |  |  |
| 20-39 | 168531 | 43.1 | 177739 | 41.7 | 176974 | 40.3 | 175645 | 38.7 | 175604 | 37.5 | 174596 | 36.4 | 170047 | 35.3 | 156941 | 34.32 |
| 40-64 | 154179 | 39.4 | 173035 | 40.6 | 183490 | 41.8 | 195550 | 43.1 | 206033 | 44.0 | 213549 | 44.5 | 214918 | 44.7 | 201929 | 44.16 |
| > 65 | 68584 | 17.5 | 75236 | 17.7 | 78738 | 17.9 | 82735 | 18.2 | 86849 | 18.5 | 91518 | 19.1 | 96247 | 20.0 | 98390 | 21.52 |
| **Gender** |  |  |  |  |  |  |  |  |  |  |  |  |  |  |  |  |
| Male | 186791 | 47.7 | 204271 | 47.9 | 210816 | 48.0 | 218397 | 48.1 | 226455 | 48.3 | 231841 | 48.3 | 231932 | 48.2 | 219905 | 48.09 |
| Female | 204503 | 52.3 | 221739 | 52.1 | 228386 | 52.0 | 235532 | 51.9 | 242030 | 51.7 | 247822 | 51.7 | 249279 | 51.8 | 237355 | 51.91 |

**S2 Table. Number of population stratified by age and gender in IADB 1999-2014**
