# Supplementary material for: Trends in polypharmacy and dispensed drugs among adults in the Netherlands as compared to the United States
Source: PLoS One. 2019 Mar 22;14(3):e0214240. doi: 10.1371/journal.pone.0214240 (PMC6430511; doi:10.1371/journal.pone.0214240)
Supplement: S3 Table — (DOCX) [file pone.0214240.s003.docx]

|  | **1999-2000** | | | | | **2001-2002** | | | | | **2003-2004** | | | | **2005-2006** | | | |
| --- | --- | --- | --- | --- | --- | --- | --- | --- | --- | --- | --- | --- | --- | --- | --- | --- | --- | --- |
|  | **No. of patients** | **Polypharmacy** | | **Prevalence % (CI)** | | **No. of patients** | **Polypharmacy** | | **Prevalence % (CI)** | | **No. of patients** | **Polypharmacy** | **Prevalence % (CI)** | | **No. of patients** | **Polypharmacy** | **Prevalence % (CI)** | |
| **Overall** | 391294 | 12076 | | 3.08  (3.03-3.14) | | 426010 | 15824 | | 3.71  (3.66-3.77) | | 439202 | 19754 | 4,50  (4.44-4.56) | | 453929 | 23039 | 5.07  (5.01-5.14) | |
| **Age group (year)** |  |  | |  | |  |  | |  | |  |  |  | |  |  |  | |
| 20-39 | 168531 | 205 | | 0.12  (0.10-0.14) | | 177739 | 272 | | 0.15  (0.13-0.17) | | 176974 | 332,5 | 0.19  (0.17-0.21) | | 175645 | 331 | 0.19  (0.17-0.21) | |
| 40-64 | 154179 | 3039 | | 1.97  (1.90-2.04) | | 173035 | 4194 | | 2.42  (2.35-2.49) | | 183490 | 5411 | 2.95  (2.87-3.03) | | 195550 | 6449 | 3.30  (3.22-3.37) | |
| > 65 | 68584 | 8832 | | 12.87  (12.65-13.12) | | 75236 | 11358 | | 15.09  (14.83-15.34) | | 78738 | 14010 | 17.79  (17.52-18.06­) | | 82735 | 16259 | 19.64  (19.37-19.91) | |
|  | **2007-2008** | | | | | **2009-2010** | | | | | **2011-2012** | | | | **2013-2014** | | | |
|  | **No. of patients** | | **Polypharmacy** | | **Prevalence % (CI)** | **No. of patients** | | **Polypharmacy** | | **Prevalence % (CI)** | **No. of patients** | **Polypharmacy** | | **Prevalence % (CI)** | **No. of patients** | **Polypharmacy** | | **Prevalence % (CI)** |
| **Overall** | 468485 | | 27298 | | 5.83  (5.76-5.89) | 479663 | | 31848 | | 6.64  (6.57-6.71) | 481211 | 36031 | | 7.49  (7.41-7.56) | 457260 | 36706 | | 8.03  (7.95-8.11) |
| **Age group (year)** |  | |  | |  |  | |  | |  |  |  | |  |  |  | |  |
| **20-39** | 175604 | | 380 | | 0.22  (0.19-0.24) | 174596 | | 419 | | 0.24  (0.22-0.26) | 170047 | 454 | | 0.27  (0.24-0.29) | 156941 | 471 | | 0.30  (0.27-0.33) |
| **40-64** | 206033 | | 7896 | | 3.83  (3.75-3.91) | 213549 | | 9250 | | 4.33  (4.24-4.42) | 214918 | 10174 | | 4.73  (4.64-4.82) | 201929 | 9988 | | 4.94  (4.85-5.04) |
| **> 65** | 86849 | | 19022 | | 21.90  (21.62-22.18) | 91518 | | 22179 | | 24.23  (23.95-24.50) | 96247 | 25404 | | 26.39  (26.12-26.67) | 98390 | 26247 | | 26.67  (26.40-26.95) |

**S3 Table. Numbers for population, polypharmacy, and polypharmacy prevalence, 1999-2014, stratified by age**
