# Supplementary material for: Trends in polypharmacy and dispensed drugs among adults in the Netherlands as compared to the United States
Source: PLoS One. 2019 Mar 22;14(3):e0214240. doi: 10.1371/journal.pone.0214240 (PMC6430511; doi:10.1371/journal.pone.0214240)
Supplement: S4 Table — Abbreviations: ACE, angiotensin-converting enzyme; COX-2, cyclooxygenase 2; NA, not applicable; NSAID, nonsteroidal anti-inflammatory drug; SSNRI, selective serotonin–norepinephrine reuptake inhibitor; SSRI, selective serotonin reuptake inhibitor. aSubgroups are presented in the order of Kantor et al. as 18 drugs classes from 1999–2012 and adjusted with the ATC codes system (see S1 Table). bExcludes COX-2 inhibitors. (DOCX) [file pone.0214240.s004.docx]

**S4 Table. Trends in prescription drug use among adults in the Netherlands 1999-2014^a^**

|  | **Prevalence of use % (95% CI)** | | | | | | | | | | | | | **2013-2014 versus**  **1999-2000** | |
| --- | --- | --- | --- | --- | --- | --- | --- | --- | --- | --- | --- | --- | --- | --- | --- |
| **Year** | **1999-2000** | | **2001-2002** | | **2003-2004** | **2005-2006** | **2007-2008** | **2009-2010** | **2011-2012** | **2013-2014** | | **P for Trend** | | **Difference Prevalence (95% CI)** | **Prevalence ratio**  **(95% CI)** |
| **Population** | **n = 391294** | | **n = 426010** | | **n = 439202** | **n = 453929** | **n = 468485** | **n = 479663** | **n = 481211** | **n = 457260** | |  |  |  |  |
| Any Prescription | 30.82  (30.68-30.96) | | 30.77  (30.63-30.91) | | 32.53  (32.39-32.67) | 31.57  (31.43-31.71) | 31.24  (31.11-31.37) | 32.06  (31.93-32.19) | 31.25  (31.12-31.38) | 31.42  (31.29-31.55) | | < 0.001 | | 0.60  (0.4-0.8) | 1.02  (1.01-1.03) |
| Polypharmacy | 3.08  (3.03-3.14) | | 3.71  (3.66-3.77) | | 4.5  (4.44-4.56) | 5.07  (5.01-5.14) | 5.83  (5.76-5.89) | 6.64  (6.57-6.71) | 7.49  (7.41-7.56) | 8.03  (7.95-8.11) | | < 0.001 | | 4.94  (4.80-5.04) | 2.60  (2.55-2.65) |
| **Antihypertensive agents** | 10.66  10.56-10.76 | | 11.50  (11.4-11.59) | | 13.99  (13.89-14.1) | 15.14  (15.04-15.25) | 16.13  (16.03-16.24) | 17.94  (17.83-18.05) | 18.85  (18.74-18.97) | 19.67  (19.55-19.78) | | < 0.001 | | 9.01  (8.85-9.15) | 1.84  (1.82-1.86) |
| Antihypertensives (other) | 0.16  (0.15-0.17) | | 0.14  (0.13-0.15) | | 0.15  (0.13-0.16) | 0.13  (0.12-0.14) | 0.12  (0.11-0.13) | 0.12  (0.11-0.13) | 0.12  (0.11-0.13) | 0.13  (0.12-0.14) | | < 0.001 | | -0.04  (-0.05)-(-0.02) | 0.78  (0.70-0.87) |
| ACE inhibitors | 1.80  (1.76-1.84) | | 1.89  (1.85-1.93) | | 2.29  (2.24-2.33) | 2.48  (2.43-2.52) | 2.70  (2.66-2.75) | 3.01  (2.97-3.06) | 3.17  (3.12-3.22) | 3.30  (3.25-3.35) | | < 0.001 | | 1.50  (1.43-1.57) | 1.83  (1.78-1.88) |
| Angiotensin II inhibitors | 0.47  (0.45-0.49) | | 0.68  (0.65-0.70) | | 1.10  (1.01-1.13) | 1.37  (1.34-1.40) | 1.60  (1.56-1.64) | 1.89  (1.85-1.93) | 2.04  (2.00-2.08) | 2.19  (2.15-2.24) | | < 0.001 | | 1.73  (1.68-1.77) | 4.69  (4.46-4.93) |
| β-blockers | 2.64  (2.59-2.69) | | 2.91  (2.86-2.96) | | 3.52  (3.46-3.57) | 3.72  (3.66-3.77) | 3.90  (3.85-3.96) | 4.33  (4.27-4.38) | 4.52  (4.46-4.57) | 4.64  (4.58-4.70) | | < 0.001 | | 2.01  (1.93-2.08) | 1.76  (1.72-1.80) |
| Calcium-channel blockers | 1.27  (1.24-1.31) | | 1.30  (1.26-1.33) | | 1.50  (1.47-1.54) | 1.51  (1.47-1.54) | 1.60  (1.56-1.64) | 1.79  (1.76-1.83) | 1.93  (1.89-1.96) | 2.14  (2.09-2.18) | | < 0.001 | | 0.87  (0.81-0.92) | 1.68  (1.63-1.74) |
| Any diuretic | 2.42  (2.37-2.47) | | 2.48  (2.43-2.53) | | 2.86  (2.82-2.91) | 3.08  (3.03-3.13) | 3.17  (3.12-3.22) | 3.43  (3.38-3.48) | 3.54  (3.48-3.59) | 3.59  (3.54-3.65) | | < 0.001 | | 1.17  (1.10-1.25) | 1.48  (1.45-1.52) |
| Loop | 0.94  (0.91-0.97) | | 0.97  (0.94-1.00) | | 1.06  (1.03-1.09) | 1.08  (1.05-1.11) | 1.08  (1.05-1.11) | 1.19  (1.16-1.22) | 1.26  (1.23-1.29) | 1.31  (1.28-1.34) | | < 0.001 | | 0.37  (0.32-0.41) | 1.39  (1.33-1.45) |
| Potassium-sparing | 0.15  (0.14-0.17) | | 0.20  (0.19-0.22) | | 0.28  (0.26-0.29) | 0.30  (0.28-0.31) | 0.32  (0.30-0.33) | 0.34  (0.32-0.35) | 0.38  (0.36-0.39) | 0.47  (0.45-0.49) | | < 0.001 | | 0.31  (0.29-0.33) | 3.04  (2.76-3.30) |
| Thiazide | 0.80  (0.78-0.83) | | 0.92  (0.89-0.95) | | 1.23  (1.19-1.26) | 1.49  (1.45-1.52) | 1.64  (1.61-1.68) | 1.83  (1.79-1.87) | 1.90  (1.86-1.94) | 1.90  (1.86-1.94) | | < 0.001 | | 1.09  (1.04-1.14) | 2.36  (2.26-2.45) |
| Antihypertensive combinations | 0 | | 0 | | 0 | 0 | 0 | 0 | 0 | 0 | | 0 | | NA | NA |
| **Antihyperlipidemic agents** | 1.52  (1.48-1.56) | | 1.83  (1.79-1.87) | | 2.56  (2.51-2.61) | 3.23  (3.18-3.28) | 3.46  (3.41-3.52) | 3.97  (3.91-4.02) | 4.31  (4.25-4.37) | 4.74  (4.68-4.80) | | < 0.001 | | 3.22  (3.14-3.29) | 3.11  (3.03-3.20) |
| Fibric acid derivatives | 0.07  (0.06-0.08) | | 0.07  (0.06-0.07) | | 0.07  (0.06-0.08) | 0.07  (0.06-0.07) | 0.06  (0.05-0.07) | 0.06  (0.05-0.07) | 0.06  (0.05-0.07) | 0.05  (0.04-0.06) | | < 0.001 | | -0.02  (-0.03)-(-0.01) | 0.70  (0.59-0.83) |
| Statins | 1.45  (1.41-1.49) | | 1.76  (1.72-1.80) | | 2.48  (2.44-2.53) | 3.11  (3.06-3.16) | 3.29  (3.24-3.35) | 3.78  (3.73-3.84) | 4.12  (4.06-4.17) | 4.54  (4.48-4.60) | | < 0.001 | | 3.09  (3.02-3.16) | 3.13  (3.03-3.22) |
| Antihyperlipidemic combinations | 0 | | 0 | | 0 | 0.02  (0.01-0.02) | 0.05  (0.04-0.05) | 0.06  (0.05-0.06) | 0.06  (0.05-0.06) | 0.06  (0.05-0.07) | | < 0.001 | | NA | NA |
| **Antidepressants** | 2.73  (2.68-2.78) | | 3.08  (3.03-3.13) | | 3.47  (3.42-3.53) | 3.46  (3.41-3.51) | 3.57  (3.52-3.62) | 3.65  (3.59-3.70) | 3.73  (3.68-3.79) | 4.01  (3.95-4.06) | | < 0.001 | | 1.28  (1.20-1.35 | 1.47  (1.43-1.50) |
| Phenylpiperazine | NA | | NA | | NA | NA | NA | NA | NA | NA | | NA | | NA | NA |
|  | | **Prevalence of use % (95% CI)** | | | | | | | | | | | **2013-2014 versus**  **1999-2000** | | |
| **Year** | | **1999-2000** | **2001-2002** | | **2003-2004** | **2005-2006** | **2007-2008** | **2009-2010** | **2011-2012** | **2013-2014** | **P for Trend** | | **Difference Prevalence (95% CI)** | | **Prevalence ratio**  **(95% CI)** |
| **Population** | | **n = 391294** | **n = 426010** | | **n = 439202** | **n = 453929** | **n = 468485** | **n = 479663** | **n = 481211** | **n = 457260** |  |  |  |  |  |
| SSNRIs | | 0.40  (0.38-0.42) | 0.52  (0.49-0.54) | | 0.67  (0.65-0.70) | 0.79  (0.76-0.81) | 0.87  (0.84-0.89) | 0.90  (0.87-092) | 0.94  (0.91-0.97) | 0.99  (0.96-1.02) | < 0.001 | | 0.59  (0.55-0.66) | | 2.46  (2.32-2.60) |
| SSRIs | | 1.43  (1.40-1.47) | 1.70  (1.66-1.74) | | 1.90  (1.86-1.94) | 1.81  (1.78-1.85) | 1.83  (1.79-1.87) | 1.80  (1.76-1.84) | 1.79  (1.75-1.83) | 1.92  (1.88-1.96) | < 0.001 | | 0.48  (0.43-0.54) | | 1.34  (1.29-1.38) |
| Tricyclics | | 0.85  (0.82-0.88) | 0.82  (0.80-0.85) | | 0.86  (0.83-0.89) | 0.80  (0.78-0.83) | 0.79  (0.76-0.81) | 0.82  (0.79-0.84) | 0.83  (0.81-0.86) | 0.90  (0.87-0.92) | 0.12 | | 0.05  (0.01-0.09) | | 1.06  (1.01-1.11) |
| **Prescription analgesics** | | 7.99  (7.91-8.08) | 8.08  (8.00-8.16) | | 8.07  (7.99-8.15) | 6.64  (6.57-6.72) | 6.58  (6.51-6.65) | 6.82  (6.75-6.89) | 6.78  (6.71-6.85) | 6.53  (6.46-6.60) | < 0.001 | | -1.46  (-1.57)-(-1.35) | | 0.82  (0.80-0.83) |
| Analgesics | | 2.31  (2.26-2.36) | 2.35  (2.30-2.40) | | 2.33  (2.29-2.38) | 1.96  (1.92-2.00) | 2.04  (2.00-2.08) | 2.17  (2.13-2.22) | 2.26  (2.22-2.31) | 2.21  (2.17-2.25) | < 0.001 | | -0.10  (-0.16)-(-0.04) | | 0.96  0.93-0.98 |
| COX-2 inhibitors | | 0.07  (0.06-0.08) | 0.24  (0.22-0.25) | | 0.59  (0.57-0.61) | 0.14  (0.13-0.15) | 0.13  (0.12-0.14) | 0.13  (0.12-0.14) | 0.14  (0.13-0.16) | 0.16  (0.15-0.17) | < 0.001 | | 0.09  (0.07-0.1) | | 2.30  (1.97-2.60) |
| Narcotic analgesics | | 0.56  (0.54-0.59) | 0.66  (0.63-0.68) | | 0.84  (0.81-0.87) | 0.94  (0.92-0.97) | 1.06  (1.03-1.09) | 1.21  (1.18-1.24) | 1.33  (1.29-1.36) | 1.35  (1.32-1.38) | < 0.001 | | 0.79  (0.74-0.83) | | 2.39  (2.28-2.51) |
| Prescription NSAIDs^b^ | | 3.44  (3.38-3.50) | 3.29  (3.24-3.34) | | 3.05  (3.00-3.10) | 2.83  (2.78-2.88) | 2.60  (2.55-2.65) | 2.58  (2.54-2.62) | 2.32  (2.28-2.36) | 2.15  (2.11-2.19) | < 0.001 | | -1.29  (-1.36)-(-1.21) | | 0.63  (0.61-0.64) |
| Salicylates | | 0.11  (0.10-0.12) | 0.09  (0.08-0.10) | | 0.07  (0.06-0.08) | 0.06  (0.05-0.07) | 0.05  (0.04-0.06) | 0.04  (0.03-0.05) | 0.04  (0.03-0.042) | 0.03  (0.02-0.03) | < 0.001 | | -0.08  (-0.09)-(-0.07) | | 0.24  (0.20-0.29) |
| Miscellaneous analgeiscs | | 1.50  (1.46-1.54) | 1.46  (1.42-1.49) | | 1.20  (1.16-1.23) | 0.71  (0.68-0.73) | 0.70  (0.67-0.72) | 0.69  (0.66-0.71) | 0.69  (0.67-0.71) | 0.63  (0.61-0.66) | < 0.001 | | -0.87  (-0.09)-(-0.07) | | 0.42  (0.40-0.44) |
| **Hormones** | | 3.35  (3.30-3.41) | 3.10  (3.05-3.15) | | 2.94  (2.89-2.99) | 2.48  (2.43-2.52) | 2.25  (2.21-2.31) | 2.30  (2.26-2.34) | 2.13  (2.09-2.17) | 2.00  (1.96-2.04) | < 0.001 | | -1.35  (-1.42)-(-1.28) | | 0.60  (0.58-0.61) |
| Sex | | 0.91  (0.88-0.94) | 0.83  (0.80-0.86) | | 0.76  (0.74-0.79) | 0.71  (0.69-0.74) | 0.65  (0.63-0.68) | 0.63  (0.61-0.65) | 0.59  (0.56-0.61) | 0.42  (0.40-0.44) | < 0.001 | | -0.49  (-0.53)-(-0.46) | | 0.46  (0.44-0.49) |
| Contraceptive | | 2.33  (2.28-2.38) | 2.16  (2.11-2.20) | | 2.12  (2.08-2.17) | 1.75  (1.71-1.78) | 1.59  (1.56-1.66) | 1.66  (1.63-1.70) | 1.55  (1.51-1.58) | 1.57  (1.54-1.61) | < 0.001 | | -0.76  (-0.81)-(-0.70) | | 0.68  (0.65-0.70) |
| Noncontraceptive | | 0.17  (0.16-0.18) | 0.16  (0.15-0.17) | | 0.08  (0.07-0.09) | 0.06  (0.05-0.06) | 0.04  (0.03-0.04) | 0.04  (0.03-0.042) | 0.03  (0.03-0.04) | 0.03  (0.03-0.04) | < 0.001 | | -0.14  (-0.15)-(-0.12) | | 0.18  (0.15-0.21) |
| **Antidiabetic agents** | | 1.51  (1.47-1.55) | 1.62  (1.58-1.66) | | 1.96  (1.92-2.00) | 2.06  (2.01-2.10) | 2.15  (2.11-2.19) | 2.45  (2.41-2.50) | 2.54  (2.49-2.58) | 2.68  (2.63-2.72) | < 0.001 | | 1.17  (1.11-1.23) | | 1.77  (1.72-1.83) |
| Biguanides | | 0.43  (0.41-0.45) | 0.56  (0.54-0.58) | | 0.78  (0.76-0.81) | 0.95  (0.92-0.98) | 1.12  (1.09-1.15) | 1.42  (1.39-1.46) | 1.60  (1.56-1.64) | 1.76  (1.72-1.79) | < 0.001 | | 1.33  (1.28-1.37) | | 4.08  (3.87-4.30) |
| Insulin | | 0.54  (0.52-0.57) | 0.58  (0.56-0.60) | | 0.70  (0.68-0.73) | 0.72  (0.70-0.75) | 0.78  (0.76-0.81) | 0.86  (0.84-0.89) | 0.83  (0.81-0.86) | 0.85  (0.83-0.88) | < 0.001 | | 0.31  (0.27-0.34) | | 1.57  (1.50-1.65) |
| Sulfonylureas | | 0.84  (0.81-0.86) | 0.83  (0.80-0.85) | | 0.90  (0.87-0.92) | 0.84  (0.81-0.87) | 0.75  (0.73-0.78) | 0.78  (0.76-0.81) | 0.79  (0.76-0.81) | 0.81  (0.79-0.84) | < 0.001 | | -0.02  (-0.06)-(-0.02) | | 0.97  (0.93-1.02) |
| Thiazolidinediones | | 0 | 0.02  (0.01-0.02) | | 0.07  (0.06-0.08) | 0.10  (0.09-0.11) | 0.06  (0.05-0.07) | 0.05  (0.04-0.06) | 0.02  (0.19-0.03) | 0.01  (0.009-0.02) | < 0.001 | | NA | | NA |
| **Prescription proton-pump inhibitors** | | 1.43  (1.40-1.47) | 1.85  (1.81-1.89) | | 2.41  (2.36-2.45) | 2.85  (2.81-2.90) | 3.56  (3.51-3.61) | 4.51  (4.45-4.57) | 5.17  (5.11-5.23) | 5.73  (5.66-5.80) | < 0.001 | | 4.30  (4.22-4.37) | | 4.00  (3.88-4.11) |
|  | |  |  | |  |  |  |  |  |  |  | |  | |  |
|  | | **Prevalence of use % (95% CI)** | | | | | | | | | | | | **2013-2014 versus**  **1999-2000** | |
| **Year** | | **1999-2000** | **2001-2002** | | **2003-2004** | **2005-2006** | **2007-2008** | **2009-2010** | **2011-2012** | **2013-2014** | | **P for Trend** | | **Difference Prevalence (95% CI)** | **Prevalence ratio**  **(95% CI)** |
| **Population** | | **n = 391294** | **n = 426010** | | **n = 439202** | **n = 453929** | **n = 468485** | **n = 479663** | **n = 481211** | **n = 457260** | |  |  |  |  |
| **Thyroid hormones** | | 0.58  (0.56-0.61) | 0.64  (0.61-0.66) | | 0.77  (0.75-0.80) | 0.83  (0.81-0.86) | 0.90  (0.88-0.93) | 1.05  (1.03-1.08) | 1.18  (1.14-1.21) | 1.32  (1.28-1.35) | | < 0.001 | | 0.73  (0.69-0.77) | 2.25  (2.15-2.36) |
| **Anxiolytics. sedatives. Hypnotics** | | 6.32  (6.24-6.40) | 6.12  (6.05-6.19) | | 6.29  (6.22-6.36) | 5.83  (5.76-5.90) | 5.52  (5.46-5.59) | 4.73  (4.67-4.79) | 4.56  (4.50-4.62) | 4.56  (4.50-4.62) | | < 0.001 | | -1.76  (-1.86)-(-1.66) | 0.72  (0.71-0.73) |
| Benzodiazepines | | 6.43  (6.35-6.51) | 6.17  (6.10-6.24) | | 6.32  (6.25-6.39) | 5.76  (5.69-5.83) | 5.38  (5.32-5.44) | 4.36  (4.30-4.42) | 4.13  (4.07-4.19) | 4.03  (3.97-4.09) | | < 0.001 | | -2.40  (-2.50)-(-2.30) | 0.63  0.61-0.64 |
| **Anticonvulsants** | | 0.50  (0.48-0.52) | 0.55  (0.53-0.57) | | 0.65  (0.63-0.67) | 0.71  (0.68-0.73) | 0.75  (0.73-0.78) | 0.82  (0.79-0.84) | 0.86  (0.83-0.88) | 0.96  (0.93-0.98) | | < 0.001 | | 0.45  (0.42-0.49) | 1.90  (1.80-2.00) |
| Benzodiazepine derivates | | 0.08  (0.07-0.09) | 0.09  (0.08-0.10) | | 0.12  (0.11-0.13) | 0.13  (0.12-0.14) | 0.13  (0.12-0.14) | 0.13  (0.12-0.14) | 0.13  (0.12-0.14) | 0.13  (0.12-0.14) | | < 0.001 | | 0.05  (0.04-0.06) | 1.63  (1.42-1.87) |
| γ-Aminobutyric acid analogs | | 0.03  (0.03-0.04) | 0.08  (0.07-0.09) | | 0.14  (0.13-0.15) | 0.21  (0.19-0.22) | 0.28  (0.26-0.29) | 0.35  (0.34-0.37) | 0.41  (0.39-0.43) | 0.51  (0.49-0.53) | | < 0.001 | | 0.48  (0.45-0.50) | 15.68  (13.02-18.58) |
| **Bronchodilators** | | 2.13  (2.09-2.18) | 2.29  (2.25-2.34) | | 2.53  (2.49-2.58) | 2.47  (2.43-2.52) | 2.50  (2.46-2.55) | 2.74  (2.69-2.78) | 2.64  (2.59-2.68) | 2.68  (2.64-2.73) | | < 0.001 | | 0.55  (0.48-0.61) | 1.26  (1.22-1.29) |
| Adrenergic bronchodilators | | 1.38  (1.35-1.42) | 1.57  (1.54-1.61) | | 1.81  (1.78-1.85) | 1.81  (1.78-1.85) | 1.87  (1.83-1.91) | 2.05  (2.01-2.09) | 1.97  (1.93-2.01) | 2.03  (1.99-2.07) | | < 0.001 | | 0.64  (0.59-0.70) | 1.47  (1.42-1.52) |
| Anticholinergic bronchodilators | | 0.51  (0.49-0.53) | 0.51  (0.49-0.54) | | 0.58  (0.55-0.60) | 0.57  (0.55-0.59) | 0.60  (0.57-0.62) | 0.68  (0.65-0.70) | 0.67  (0.65-0.69) | 0.70  (0.68-0.73) | | < 0.001 | | 0.19  (0.16-0.23) | 1.38  (1.30-1.46) |
| Bronchodilator combinations | | 0.17  (0.16-0.19) | 0.45  (0.43-0.47) | | 0.85  (0.82-0.87) | 1.05  (1.02-1.08) | 1.17  (1.14-1.20) | 1.35  (1.32-1.38) | 1.34  (1.30-1.37) | 1.38  (1.34-1.41) | | < 0.001 | | 1.20  (1.17-1.24) | 8.00  (7.37-8.64) |
| **Antibiotics** | |  |  | |  |  |  |  |  |  | |  | |  |  |
| Oral antibiotics | | 2.69  (2.64-2.74) | 2.68  (2.63-2.73) | | 2.96  (2.91-3.01) | 2.97  (2.92-3.02) | 2.98  (2.93-3.03) | 3.23  (3.18-3.28) | 2.80  (2.75-2.84) | 2.73  (2.68-2.78) | | < 0.001 | | 0.04  (0.03-0.11) | 1.01  (0.99-1.04) |
| **Antiarrhythmic agents** | | 1.19  (1.16-1.22) | 1.08  (1.05-1.11) | | 1.06  (1.03-1.09) | 0.90  (0.87-0.93) | 0.80  (0.77-0.83) | 0.80  (0.77-0.83) | 0.78  (0.75-0.80) | 0.79  (0.76-0.82) | | < 0.001 | | -0.40  (-0.46)-(-0.37) | 0.66  (0.63-0.68) |
| Class I and III | | 0.17  (0.16-0.18) | 0.18  (0.17-0.19) | | 0.17  (0.16-0.18) | 0.16  (0.15-0.17) | 0.16  (0.15-0.17) | 0.15  (0.14-0.16) | 0.15  (0.14-0.16) | 0.15  (0.14-0.16) | | < 0.001 | | -0.02  (-0.04)-(-0.01) | 0.88  (0.77-0.95) |
| Class IV | | 0.56  (0.54-0.59) | 0.50  (0.48-0.53) | | 0.51  (0.48-0.53) | 0.42  (0.40-0.44) | 0.37  (0.35-0.39) | 0.36  (0.34-0.38) | 0.33  (0.32-0.35) | 0.34  (0.33-0.36) | | < 0.001 | | -0.22  (-0.26)-(-0.00) | 0.61  (0.56-0.64) |
| Class V | | 0.46  (0.44-0.48) | 0.40  (0.38-0.42) | | 0.38  (0.36-0.40) | 0.32  (0.30-0.34) | 0.27  (0.26-0.28) | 0.29  (0.27-0.31) | 0.30  (0.28-0.32) | 0.30  (0.28-0.32) | | < 0.001 | | -0.16  (-0.19)-(-0.14) | 0.65  (0.60-0.69) |
| **Coagulation modifiers** | | 2.86  (2.81-2.91) | 2.94  (2.89-2.99) | | 3.46  (3.41-3.51) | 3.60  (3.55-3.65) | 3.77  (3.72-3.82) | 4.15  (4.09-4.21) | 4.24  (4.18-4.30) | 4.52  (4.46-4.58) | | < 0.001 | | 1.66  (1.58-1.74) | 1.58  (1.54-1.61) |
| Anticoagulants | | 2.81  (2.76-2.86) | 2.90  (2.85-2.95) | | 3.42  (3.37-3.48) | 3.58  (3.53-3.63) | 3.75  (3.69-3.80) | 4.13  (4.08-4.19) | 4.22  (4.16-4.28) | 4.49  (4.43-4.55) | | < 0.001 | | 1.68  (1.60-1.76) | 1.60  (1.56-1.63) |
| Warfarin | | 0 | 0 | | 0 | 0 | 0.00011  (0.00-0.00) | 0 | 0 | 0 | | NA | | NA | NA |
| Antiplatelet agents | | 2.05  (2.01-2.10) | 2.16  (2.11-2.20) | | 2.53  (2.49-2.58) | 2.62  (2.58-2.67) | 2.71  (2.67-2.76) | 3.03  (2.99-3.08) | 3.14  (3.09-3.19) | 3.27  (3.22-3.32) | | < 0.001 | | 1.22  (1.15-1.29) | 1.59  (1.55-1.64) |
|  | |  |  | |  |  |  |  |  |  | |  | |  |  |
|  | | **Prevalence of use % (95% CI)** | | | | | | | | | | | | **2013-2014 versus**  **1999-2000** | |
| **Year** | | **1999-2000** | | **2001-2002** | **2003-2004** | **2005-2006** | **2007-2008** | **2009-2010** | **2011-2012** | **2013-2014** | | **P for Trend** | | **Difference Prevalence (95% CI)** | **Prevalence ratio (95% CI)** |
| **Population** | | **n = 391294** | | **n = 426010** | **n = 439202** | **n = 453929** | **n = 468485** | **n = 479663** | **n = 481211** | **n = 457260** | |  |  |  |  |
| Clopidogrel | | 0.007  (0.004-0.009) | | 0.03  (0.03-0.04) | 0.08  (0.07-0.09) | 0.13  (0.11-0.14) | 0.16  (0.14-0.17) | 0.21  (0.20-0.22) | 0.22  (0.20-0.23) | 0.21  (0.19-0.22) | | < 0.001 | | 0.20  (0.18-0.21) | 31.05  (20.88-45.53) |
| **Muscle relaxants** | | 0.04  (0.04-0.05) | | 0.04  (0.03-0.047) | 0.04  (0.038-0.05) | 0.05  (0.04-0.053) | 0.05  (0.04-0.06) | 0.05  (0.05-0.06) | 0.06  (0.06-0.07) | 0.07  (0.06-0.08) | | < 0.001 | | 0.03  (0.02-0.04) | 1.59  (1.32-1.92) |
| **Nasal preparations** | | 0.80  (0.77-0.83) | | 0.90  (0.87-0.92) | 0.98  (0.95-1.01) | 0.92  (0.89-0.95) | 1.00  (0.97-1.03) | 1.21  (1.18-1.24) | 1.21  (1.18-1.24) | 1.28  (1.25-1.32) | | < 0.001 | | 0.49  (0.44-0.53) | 1.61  (1.54-1.68) |
| Nasal steroids | | 0.65  (0.62-0.68) | | 0.75  (0.72-0.78) | 0.86  (0.83-0.89) | 0.84  (0.81-0.87) | 0.91  (0.88-0.94) | 1.12  (1.09-1.15) | 1.13  (1.10-1.16) | 1.21  (1.18-1.24) | | < 0.001 | | 0.56  (0.52-0.61) | 1.86  (1.79-1.96) |
| **H2 Antagonists** | | 0.90  (0.87-0.93) | | 0.74  (0.72-0.77) | 0.66  (0.64-0.69) | 0.48  (0.46-0.50) | 0.35  (0.33-0.36) | 0.29  (0.28-0.31) | 0.23  (0.22-0.25) | 0.22  (0.20-0.23) | | < 0.001 | | -0.68  (-0.71)-(-0.65) | 0.24  (0.22-0.36) |
| **Prescription antihistamines** | | 0.84  (0.81-0.87) | | 0.89  (0.86-0.92) | 0.96  (0.93-0.99) | 0.96  (0.93-0.99) | 0.97  (0.94-1.00) | 1.03  (1.01-1.06) | 1.04  (1.01-1.07) | 1.09  (1.06-1.12) | | < 0.001 | | 0.26  (0.21-0.30) | 1.30  (1.25-1.36) |

Abbreviations: ACE, angiotensin-converting enzyme; COX-2, cyclooxygenase 2; NA, not applicable; NSAID, nonsteroidal anti-inflammatory drug; SSNRI, selective serotonin–norepinephrine reuptake inhibitor;SSRI, selective serotonin reuptake inhibitor. ^a^Subgroups are presented in the order of Kantor *et al.* as 18 drugs classes from 1999-2012 and adjusted with the ATC codes system (see S1 Table). ^b^Excludes COX-2 inhibitors
