# Supplementary material for: Trends in polypharmacy and dispensed drugs among adults in the Netherlands as compared to the United States
Source: PLoS One. 2019 Mar 22;14(3):e0214240. doi: 10.1371/journal.pone.0214240 (PMC6430511; doi:10.1371/journal.pone.0214240)
Supplement: S5 Table — Abbreviations: ACE, angiotensin-converting enzyme; COX-2, cyclooxygenase 2; NA, not assessed; NSAID, nonsteroidal anti-inflammatory drug; SSNRI, selective serotonin–norepinephrine reuptake inhibitor; SSRI, selective serotonin reuptake inhibitor.aSubgroups are presented in the order of Kantor et al. as 18 drugs classes from 1999–2012 and adjusted with the ATC codes system (see S1 Table). bExcludes COX-2 inhibitors. (DOCX) [file pone.0214240.s005.docx]

**S5 Table. Prevalence of prescription drug use among adults in the Netherlands^a^**

|  | **Prevalence of use, %, (95%CI)** | | **Difference Prevalence (95% CI)** | **Prevalence ratio**  **(95% CI)** |
| --- | --- | --- | --- | --- |
|  | **The Netherlands 1999-2000** | **The Netherlands 2011-2012** |  |  |
|  | **n = 391294** | **n = 481211** |  |  |
| Any Prescription | 30.82 (30.68-30.96) | 31.25 (31.12-31.38) | 0.43 (0.20-0.60) | 1.01 (1.00-1.02) |
| Polypharmacy | 3.08 (3.03-3.14) | 7.49 (7.41-7.56) | 4.40 (4.32-4.48) | 2.43 (2.38-2.47) |
| **Antihypertensive agents** | 10.66 10.56-10.76 | 18.85 (18.74-18.97) | 8.19 (8.01-9.34) | 1.77 (1.75-1.78) |
| Antihypertensives (other) | 0.16 (0.15-0.17) | 0.12 (0.11-0.13) | -0.04 (-0.05)-(-0.02) | 0.77 (0.69-0.86) |
| ACE inhibitors | 1.80 (1.76-1.84) | 3.17 (3.12-3.22) | 1.37 (1.30-1.43) | 1.76 (1.71-1.81) |
| Angiotensin II inhibitors | 0.47 (0.45-0.49) | 2.04 (2.00-2.08) | 1.58 (1.53-1.62) | 4.37 (4.15-4.58) |
| β-blockers | 2.64 (2.59-2.69) | 4.52 (4.46-4.57) | 1.88 (1.80-1.96) | 1.71 (1.67-1.75) |
| Calcium-channel blockers | 1.27 (1.24-1.31) | 1.93 (1.89-1.96) | 0.66 (0.60-0.71) | 1.52 (1.46-1.57) |
| Any diuretic | 2.42 (2.37-2.47) | 3.54 (3.48-3.59) | 1.11 (1.04-1.19) | 1.46 (1.42-1.50) |
| Loop | 0.94 (0.91-0.97) | 1.26 (1.23-1.29) | 0.32 (0.27-0.36) | 1.33 (1.28-1.39) |
| Potassium-sparing | 0.15 (0.14-0.17) | 0.38 (0.36-0.39) | 0.22 (0.20-0.24) | 2.45 (2.23-2.68) |
| Thiazide | 0.80 (0.78-0.83) | 1.90 (1.86-1.94) | 1.10 (1.05.-1.14) | 2.36 (2.27-2.46) |
| Antihypertensive combinations | 0 | 0 | NA | NA |
| **Antihyperlipidemic agents** | 1.52 (1.48-1.56) | 4.31 (4.25-4.37) | 2.79 (2.72-2.86) | 2.83 (2.75-2.91) |
| Fibric acid derivatives | 0.07 (0.06-0.08) | 0.06 (0.05-0.06) | -0.02 (-0.03)-(-0.01) | 0.78 (0.66-0.92) |
| Statins | 1.45 (1.41-1.49) | 4.12 (4.06-4.17) | 2.66 (2.60-2.73) | 2.83 (2.75-2.92) |
| Antihyperlipidemic combinations | 0 | 0.06 (0.05-0.06) | NA | NA |
| **Antidepressants** | 2.73 (2.68-2.78) | 3.73 (3.68-3.79) | 1.01 (0.93-1.08) | 1.37 (1.34-1.40) |
| Phenylpiperazine | NA | NA | NA | NA |
| SSNRIs | 0.40 (0.38-0.42) | 0.94 (0.91-0.97) | 0.54 (0.50-0.57) | 2.33 (2.20-2.46) |
| SSRIs | 1.43 (1.40-1.47) | 1.79 (1.75-1.83) | 0.36 (0.30-0.41) | 1.25 (1.21-1.29) |
| Tricyclics | 0.85 (0.82-0.88) | 0.83 (0.81-0.86) | -0.02 (-0.05)-(-0.01) | 0.98 (0.94-1.01) |
| **Prescription analgesics** | 7.99 (7.91-8.08) | 6.78 (6.71-6.85) | -1.21 (-1.32)-(-1.09) | 0.85 (0.84-0.86) |
| Analgesics | 2.31 (2.26-2.36) | 2.26 (2.22-2.31) | -0.05 (-0.11)-(-0.02) | 0.98 (0.95-1.01) |
| COX-2 inhibitors | 0.07 (0.06-0.08 | 0.14 (0.13-0.16) | 0.07 (0.06-0.09) | 2.06 (1.79-2.37) |
| Narcotic analgesics | 0.56 (0.54-0.59) | 1.33 (1.29-1.36) | 0.76 (0.72-0.80) | 2.35 (2.23-2.46) |
|  | **Prevalence of use, %, (95%CI)** | | **Difference Prevalence (95% CI)** | **Prevalence ratio**  **(95% CI)** |
|  | **The Netherlands 1999-2000** | **The Netherlands 2011-2012** |  |  |
|  | **n = 391294** | **n = 481211** |  |  |
| Prescription NSAIDs^b^ | 3.44 (3.38-3.50) | 2.32 (2.28-2.36) | -1.12 (-1.19)-(-1.04) | 0.67 (0.66-0.69) |
| Salicylates | 0.11 (0.10-0.12) | 0.04 (0.03-0.04) | -0.07 (-0.08)-(-0.06) | 0.34 (0.29-0.41) |
| Miscellaneous analgeiscs | 1.50 (1.46-1.54) | 0.69 (0.67-0.71) | -0.81 (-0.86)-(-0.77) | 0.46 (0.44-0.48) |
| **Hormones** | 3.35 (3.30-3.41) | 2.13 (2.09-2.17) | -1.22 (-1.29)-(-1.15) | 0.64 (0.62-0.65) |
| Sex | 0.91 (0.88-0.94) | 0.59 (0.56-0.61) | -0.33 (-0.36)-(-0.29) | 0.64 (0.61-0.68) |
| Contraceptive | 2.33 (2.28-2.38) | 1.55 (1.51-1.58) | -0.78 (-0.84)-(-0.72) | 0.66 (0.64-0.68) |
| Noncontraceptive | 0.17 (0.16-0.18) | 0.03 (0.03-0.04) | -0.14 (-0.15)-(-0.12) | 0.18 (0.15-0.22) |
| **Antidiabetic agents** | 1.51 (1.47-1.55) | 2.54 (2.49-2.58) | 1.03 (0.97-1.09) | 1.68 (1.63-1.73) |
| Biguanides | 0.43 (0.41-0.45) | 1.60 (1.56-1.64) | 1.17 (1.13-1.21) | 3.72 (1.13-1.21) |
| Insulin | 0.54 (0.52-0.57) | 0.83 (0.81-0.86) | 0.29 (0.26-0.33) | 1.54 (1.46-1.62) |
| Sulfonylureas | 0.84 (0.81-0.86) | 0.79 (0.76-0.81) | -0.05 (-0.09)-(-0.01) | 0.94 (0.90-0.99) |
| Thiazolidinediones | 0 | 0.02 (0.02-0.03) | NA | NA |
| **Prescription proton-pump inhibitors** | 1.43 (1.40-1.47) | 5.17 (5.11-5.23) | 3.74 (3.66-3.81) | 3.61 (3.50-3.71) |
| **Thyroid hormones** | 0.58 (0.56-0.61) | 1.18 (1.14-1.21) | 0.59 (0.55-0.63) | 2.01 (1.92-2.11) |
| **Anxiolytics, sedatives, hypnotics** | 6.32 (6.24-6.40) | 4.56 (4.50-4.62) | -1.76 (-1.85)-(-1.66) | 0.72 (0.71-0.74) |
| Benzodiazepines | 6.43 (6.35-6.51) | 4.13 (4.07-4.19) | -2.30 (-2.39)-(-2.20) | 0.64 (0.63-0.65) |
| **Anticonvulsants** | 0.50 (0.48-0.52) | 0.86 (0.83-0.88) | 0.35 (0.32-0.39) | 1.70 (1.61-1.79) |
| Benzodiazepine derivates | 0.08 (0.07-0.09) | 0.13 (0.12-0.14) | 0.05 (0.03-0.06) | 1.59 (1.39-1.83) |
| γ-Aminobutyric acid analog | 0.03 (0.03-0.04) | 0.41 (0.39-0.43) | 0.38 (0.36-0.40) | 12.63 (10.45-15.00) |
| **Bronchodilators** | 2.13 (2.09-2.18) | 2.64 (2.59-2.68) | 0.50 (0.44-0.57) | 1.23 (1.20-1.27) |
| Adrenergic bronchodilators | 1.38 (1.35-1.42) | 1.97 (1.93-2.01) | 0.59 (0.53-0.64) | 1.43 (1.38-1.47) |
| Anticholinergic bronchodilators | 0.51 (0.49-0.53) | 0.67 (0.65-0.69) | 0.16 (0.13-0.20) | 1.32 (1.25-1.40) |
| Bronchodilator combinations | 0.17 (0.16-0.19) | 1.34 (1.30-1.37) | 1.16 (1.13-1.20) | 7.76 (7.15-8.38) |
| **Antibiotics** | NA | NA | NA | NA |
| Oral antibiotics | 2.69 (2.64-2.74) | 2.80 (2.75-2.84) | 0.10 (0.04-0.17) | 1.04 (1.01-1.06) |
| **Antiarrhythmic agents** | 1.90 (1.86-1.94) | - 1. (2.33-2.42) | -0.41 (-0.46)-(-0.37) | 0.66 (0.63-0.68) |

|  | **Prevalence of Use, %, (95%CI)** | | **Difference Prevalence (95% CI)** | **Prevalence ratio**  **(95% CI)** |
| --- | --- | --- | --- | --- |
|  | **The Netherlands 1999-2000** | **The Netherlands 2011-2012** |  |  |
|  | **n = 391294** | **n = 481211** |  |  |
| Class I and III | 0.17 (0.16-0.18) | 0.15 (0.14-0.16) | -0.02 (-0.04)-(-0.01) | 0.88 (0.78-0.97) |
| Class IV | 0.56 (0.54-0.59) | 0.33 (0.32-0.35) | -0.16 (-0.18)-(-0.13) | 0.59 (0.55-0.62) |
| Class V | 0.46 (0.44-0.48) | 0.30 (0.28-0.32) | -0.16 (-0.18)-(-0.13) | 0.65 (0.62-0.70) |
| **Coagulation modifiers** | 2.86 (2.81-2.91) | 4.24 (4.18-4.30) | 1.38 (1.31-1.46) | 1.48 (1.45-1.52) |
| Anticoagulants | 2.81 (2.76-2.86) | 4.22 (4.16-4.28) | 1.41 (1.33-1.49) | 1.50 (1.47-1.54) |
| Warfarin | NA | NA | NA | NA |
| Antiplatelet agents | 2.05 (2.01-2.10) | 3.14 (3.09-3.19) | 1.08 (1.00-1.13) | 1.53 (1.48-1.56) |
| Clopidogrel | 0.007 (0.004-0.009) | 0.22 (0.20-0.23) | 0.21 (0.20-0.22) | 32.62 (22.06-48.04) |
| **Muscle relaxants** | 0.04 (0.038-0.05) | 0.06 (0.057-0.07) | 0.02 (0.01-0.03) | 1.44 (1.20-1.73) |
| **Nasal preparations** | 0.80 (0.77-0.83) | 1.21 (1.18-1.24) | 0.41 (0.37-0.45) | 1.52 (1.45-1.58) |
| Nasal steroids | 0.65 (0.62-0.68) | 1.13 (1.10-1.16) | 0.48 (0.45-0.52) | 1.74 (1.67-1.83) |
| **H2 Antagonist** | 0.90 (0.87-0.93) | 0.23 (0.22-0.25) | -0.66 (-0.69)-(-0.63) | 0.26 (0.24-0.28) |
| **Prescription antihistamines** | 0.84 (0.81-0.84) | 1.04 (1.01-1.07) | 0.21 (0.16-0.25) | 1.24 (1.19-1.30) |

Abbreviations: ACE, angiotensin-converting enzyme; COX-2, cyclooxygenase 2; NA, not assessed; NSAID, nonsteroidal anti-inflammatory drug; SSNRI, selective serotonin–norepinephrine reuptake inhibitor; SSRI, selective serotonin reuptake inhibitor. ^a^Subgroups are presented in the order of Kantor *et al.* as 18 drugs classes from 1999-2012 and adjusted with the ATC codes system (See S1 Table). ^b^Excludes COX-2 inhibitors.
