# Supplementary material for: Trends in polypharmacy and dispensed drugs among adults in the Netherlands as compared to the United States
Source: PLoS One. 2019 Mar 22;14(3):e0214240. doi: 10.1371/journal.pone.0214240 (PMC6430511; doi:10.1371/journal.pone.0214240)
Supplement: S6 Table — (DOCX) [file pone.0214240.s006.docx]

**S6 Table. STROBE Statement—checklist of items that should be included in reports of observational studies**

|  | Item No. | Recommendation | Page  No. | Relevant text from manuscript |
| --- | --- | --- | --- | --- |
| **Title and abstract** | 1 | (*a*) Indicate the study’s design with a commonly used term in the title or the abstract | 2 | We conducted a repeated cross-sectional study using the Dutch IADB.nl prescription database. |
|  |  | (*b*) Provide in the abstract an informative and balanced summary of what was done and what was found | 2 | Polypharmacy more than doubled from 1999 to 2014, and this increase was not limited to the elderly. The relative increase was larger in the Netherlands compared to the US, which was partly due to larger increases in several guideline-recommended preventive drugs |
| Introduction | | | | |
| Background/rationale | 2 | Explain the scientific background and rationale for the investigation being reported | 5 | Knowledge about trends in polypharmacy, and the age groups and drugs which contribute to these trends, is important to determine whether efforts to reduce polypharmacy may be needed |
| Objectives | 3 | State specific objectives, including any prespecified hypotheses | 5 | Our aim is to obtain insights into the trends in polypharmacy stratified by age and the underlying drugs among adults in the Netherlands between 1999 and 2014. |
| Methods | | | | |
| Study design | 4 | Present key elements of study design early in the paper | 5 | We conducted a repeated cross-sectional study to assess trends in polypharmacy and dispensed drugs among adults in the Netherlands from 1 January 1999 until 31 December 2014 using data from a population-based prescription database (IADB.nl). |
| Setting | 5 | Describe the setting, locations, and relevant dates, including periods of recruitment, exposure, follow-up, and data collection | 5-6 | In part of Methods section:  Study Design  Data Source  Study Population |
| Participants | 6 | (*a*) *Cohort study*—Give the eligibility criteria, and the sources and methods of selection of participants. Describe methods of follow-up  *Case-control study*—Give the eligibility criteria, and the sources and methods of case ascertainment and control selection. Give the rationale for the choice of cases and controls  ***Cross-sectional study*—Give the eligibility criteria, and the sources and methods of selection of participants** | 5-6 | In part of Methods section:  Study Design  Data Source  Study Population |
|  |  | (*b*) *Cohort study*—For matched studies, give matching criteria and number of exposed and unexposed  *Case-control study*—For matched studies, give matching criteria and the number of controls per case | N/A |  |
| Variables | 7 | Clearly define all outcomes, exposures, predictors, potential confounders, and effect modifiers. Give diagnostic criteria, if applicable | 6-7 | In part of Methods section:  Polypharmacy Definition  Outcomes |
| Data sources/ measurement | 8* | For each variable of interest, give sources of data and details of methods of assessment (measurement). Describe comparability of assessment methods if there is more than one group | N/A |  |
| Bias | 9 | Describe any efforts to address potential sources of bias | N/A |  |
| Study size | 10 | Explain how the study size was arrived at | N/A |  |

Continued on next page

| Quantitative variables | | 11 | Explain how quantitative variables were handled in the analyses. If applicable, describe which groupings were chosen and why | 7 | | In part of Methods section: Analysis | |
| --- | --- | --- | --- | --- | --- | --- | --- |
| Statistical methods | | 12 | (*a*) Describe all statistical methods, including those used to control for confounding | 7 | | Linear-by-linear association in the Chi-square analysis was used to calculate the p-value for the prevalence rates trend. Statistical significance was assessed at the 2-sided α = 0.05 level | |
|  |  |  | (*b*) Describe any methods used to examine subgroups and interactions | 7 | | We ranked the prevalences of the drug groups at the ATC2 level to identify the 20 most commonly dispensed drug groups for each two-year period. We calculated the absolute differences and prevalence ratios for these drug groups between the first and the last two-year period. The prevalence ratio indicates the relative increase or decrease in prevalence over the study period. To compare trends in the Netherlands with the US, we calculated the prevalence ratios for polypharmacy and for 18 drug groups – as presented by Kantor et al. – between 1999-2000 and 2011-2012 | |
|  |  |  | (*c*) Explain how missing data were addressed | N/A | |  | |
|  |  |  | (*d*) *Cohort study*—If applicable, explain how loss to follow-up was addressed  *Case-control study*—If applicable, explain how matching of cases and controls was addressed  *Cross-sectional study*—If applicable, describe analytical methods taking account of sampling strategy | N/A | |  | |
|  |  |  | (*e*) Describe any sensitivity analyses | N/A | |  | |
| Results | | | | | | | |
| Participants | | 13* | (a) Report numbers of individuals at each stage of study—eg numbers potentially eligible, examined for eligibility, confirmed eligible, included in the study, completing follow-up, and analysed | Fig 1 | | | The number of patients included in this study from 1999 to 2014 were 391,294, 426,010, 439,202, 453,929, 468,485, 479,663, 481,211 and 457,260, respectively (Fig 1). The populations stratified by age and gender are shown in S2 Table. |
|  |  |  | (b) Give reasons for non-participation at each stage | Fig 1 | | |  |
|  |  |  | (c) Consider use of a flow diagram | Fig 1 | | |  |
| Descriptive data | | 14* | (a) Give characteristics of study participants (eg demographic, clinical, social) and information on exposures and potential confounders | S2 Table | | |  |
|  |  |  | (b) Indicate number of participants with missing data for each variable of interest | N/A | | |  |
|  |  |  | (c) *Cohort study*—Summarise follow-up time (eg, average and total amount) | N/A | | |  |
| Outcome data | | 15* | *Cohort study*—Report numbers of outcome events or summary measures over time |  | | |  |
|  |  |  | *Case-control study—*Report numbers in each exposure category, or summary measures of exposure |  | | |  |
|  |  |  | ***Cross-sectional study—*Report numbers of outcome events or summary measures** | 8-10 | | | Part of Result section:  Trends in polypharmacy in the Netherlands 1999-2014  Trends in Drug Groups in the Netherlands 1999-2014  Comparison of trends with the United States |
| Main results | | 16 | (*a*) Give unadjusted estimates and, if applicable, confounder-adjusted estimates and their precision (eg, 95% confidence interval). Make clear which confounders were adjusted for and why they were included | 8 | | | The two-year polypharmacy prevalence rates increased from 3.08% to 8.03% in the study period (p-value for trend <0.001). When stratified by age group, the prevalence of polypharmacy increased significantly for all age groups (Fig. 2 and S3 Table). |
|  |  |  | (*b*) Report category boundaries when continuous variables were categorized | N/A | | |  |
|  |  |  | (*c*) If relevant, consider translating estimates of relative risk into absolute risk for a meaningful time period | Table 1, S4, S5 Tables | | |  |
| Other analyses | 17 | Report other analyses done—eg analyses of subgroups and interactions, and sensitivity analyses | | Table 1, S4, S5 Tables | | |  |
| Discussion | | | | | | | |
| Key results | 18 | Summarise key results with reference to study objectives | | 13 | The prevalence of polypharmacy among adults in the Netherlands between 1999 and 2014 increased more than twofold, from around 3% to 8% in the overall population. Increases were observed in all age groups, but the relative increases were larger in the under-65 age groups. Preventive therapies showed the highest prevalence rates, and the largest increases were seen for proton-pump inhibitors, angiotensin-II inhibitors and statins. In general, the increase in prevalence rate was lower in the Netherlands compared to the US, but similar trends were observed for many drugs. | | |
| Limitations | 19 | Discuss limitations of the study, taking into account sources of potential bias or imprecision. Discuss both direction and magnitude of any potential bias | | 17 | Some limitions need to be mentioned. First, information about the actual use of drugs by the patients was unavailable because we only looked at the drug prescriptions being dispensed. We do not know whether patients actually took the drugs they were prescribed. We also do not know which OTC drugs the patients used. In the Netherlands, OTC drugs include drugs for common complaints, such as pain, cough, hay fever, itching, and stomach complaints. Second, for the polypharmacy calculation we counted drugs at the pharmacological group level (ATC3) in the last four months of each year, as proposed in the Dutch guidelines for polypharmacy. This assumes that drugs within this level are similar and usually not combined for chronic use. This prevents switches between drugs at this level in the assessment period from being double counted. Combination drugs at this level, however, were counted as a single drug, which could cause underestimation of the polypharmacy prevalence. Third, we used aggregated data when testing for trends and were therefore unable to adjust for correlations at patient level over time. Different methods were used for estimating prescription rates in our study as compared to the US study. | | |
| Interpretation | 20 | Give a cautious overall interpretation of results considering objectives, limitations, multiplicity of analyses, results from similar studies, and other relevant evidence | | 19 | The prevalence of polypharmacy in the Netherlands has increased significantly from 1999 to 2014. This increase was not limited to the elderly, and may even have halted in the elderly in 2013-2014. Increases in the underlying drugs partly relate to changed guideline recommendations and reimbursement policies, but this does not exclude the possibility of overtreatment, particularly in the elderly. Despite differences in the methods used to assess prescription drug use, increases in prevalence rates are lower in the Netherlands than in the US. Over time the differences in prevalence rates have become smaller. | | |
| Generalisability | 21 | Discuss the generalisability (external validity) of the study results | | 18 | Increases and decreases seem to relate to changes in the policies for certain drugs, such as in the reimbursement system or prescription policy. Some trends, however, might be worrying. Where strict management of cardiovascular risk factors is advocated in general, concerns are growing about overtreatment in the elderly population [51]. The increase in these drugs may be appropriate in the groups aged under 65, on the other hand. More detailed analyses are thus warranted in this younger population. Commonly used tools, such as the START/STOPP criteria, have been developed for people aged 65 and older. Therefore, there is a need for similar criteria which can be applied in younger age groups. For example, following from our results, this could include potentially inappropriate use of PPIs. New reports about serious adverse events have informed more critical views regarding the increasing use of these drugs and indications for chronic use in the younger age groups are limited [34,52].  Complex interventions are needed to manage polypharmacy and halt rising trends in the use of potentially inappropriate drugs, such as advanced medication reviews and medication deprescribing [23]. Constant monitoring of prescription drug use is warranted to assess the effects of policy measures and signal potential inappropriate drug use. | | |
| Other information | |  | | | | | |
| Funding | 22 | Give the source of funding and the role of the funders for the present study and, if applicable, for the original study on which the present article is based | | N/A |  | | |

*Give information separately for cases and controls in case-control studies and, if applicable, for exposed and unexposed groups in cohort and cross-sectional studies.

**Note:** An Explanation and Elaboration article discusses each checklist item and gives methodological background and published examples of transparent reporting. The STROBE checklist is best used in conjunction with this article (freely available on the Web sites of PLoS Medicine at http://www.plosmedicine.org/, Annals of Internal Medicine at http://www.annals.org/, and Epidemiology at http://www.epidem.com/). Information on the STROBE Initiative is available at www.strobe-statement.org.
